# RARα supports the development of Langerhans cells and langerin-expressing conventional dendritic cells

**DOI:** 10.1038/s41467-018-06341-8

**Published:** 2018-09-25

**Authors:** Seika Hashimoto-Hill, Leon Friesen, Sungtae Park, Suji Im, Mark H. Kaplan, Chang H. Kim

**Affiliations:** 1 Department of Comparative Pathobiology, Purdue University, West Lafayette, IN 47907 USA; 2 Laboratory of Immunology and Hematopoiesis, Mary H. Weiser Food Allergy Center, University of Michigan Medical School, Ann Arbor, MI 48109 USA; 3 Department of Pathology, University of Michigan Medical School, Ann Arbor, MI 48109 USA; 4 Department of Pediatrics, Indiana University School of Medicine, Indianapolis, IN 46202 USA

## Abstract

Langerhans cells (LC) are the prototype langerin-expressing dendritic cells (DC) that reside specifically in the epidermis, but langerin-expressing conventional DCs also reside in the dermis and other tissues, yet the factors that regulate their development are unclear. Because retinoic acid receptor alpha (RARα) is highly expressed by LCs, we investigate the functions of RARα and retinoic acid (RA) in regulating the langerin-expressing DCs. Here we show that the development of LCs from embryonic and bone marrow-derived progenitors and langerin^+ ^conventional DCs is profoundly regulated by the RARα-RA axis. During LC differentiation, RARα is required for the expression of a LC-promoting transcription factor Runx3, but suppresses that of LC-inhibiting C/EBPβ. RARα promotes the development of LCs and langerin^+^ conventional DCs only in hypo-RA conditions, a function effectively suppressed at systemic RA levels. Our findings identify positive and negative regulatory mechanisms to tightly regulate the development of the specialized DC populations.

## Abstract (web summary)

Langerhans cells (LC) and langerin-expressing conventional dendritic cells are made from distinct progenitors and enriched in the distinct microenvironments of the skin. Here the authors show that these immune cells are regulated by retinoic acid receptor alpha (RARα) via simultaneous induction of LC-promoting Runx3 and repression of LC-inhibiting C/EBPβ.

## Introduction

Langerhans cells (LCs) are the prototype dendritic cells that reside specifically in the epidermis. At steady state, LCs are the only MHC-II-expressing antigen-presenting cells in the epidermis. Langerin^+^ conventional dendritic cells (cDCs), similar to LCs, are also found in other tissues, including dermis, lymph nodes, spleen and lungs, albeit at significantly lower frequencies. A long-standing question is how LC development occurs selectively in the epidermis.

The developmental origin of LCs is different from that of cDCs. LCs are developed from embryonic myeloid precursors from the yolk sac and fetal liver, and fully differentiated langerin^+^ LCs appear within a few days following birth in mice^1–4^. These cells can self-renew and persist in the skin throughout the life^5^. However, the LCs of embryonic origin can be replaced by bone marrow (BM)-derived LCs in inflammatory conditions^6^. Other langerin^+^ cDCs are thought to be generated from BM-derived precursors^7,8^. LC development is positively regulated by two cytokines, TGF-β and IL-34^9–15^. LC development is promoted by certain transcription factors, such as PU.1, inhibitor of DNA binding 2 (Id2) and runt-related transcription factor 3 (Runx3), and suppressed by C/EBPβ (CCAAT/enhancer-binding protein β)^16–18^. Tissue factors that tightly control the development of LC and langerin^+^ cDCs in the body remain unclear.

Retinoic acids (RAs) and their receptors play pivotal roles in embryo morphogenesis and immune regulation^19,20^. RA influences myeloid cell differentiation^21,22^ and generates mucosal DCs that express retinal aldehyde dehydrogenase 2 (RALDH2), Arg1, and gut-homing receptors^23–28^. It is also reported that RA affects pre-DC differentiation into CD11b^+^CD8α^-^ vs. CD11b^-^CD8α^+^ subsets, expanding the former subset in the spleen^29,30^. Vitamin A deficiency (VAD) decreases the size of the intestinal CD103^+^CD11b^+^ DC population^29,30^, but expands langerin^+^ DCs in mucosal tissues^31,32^. However, the role of RA in regulating LC differentiation is not established.

Here we report that the development of LCs and langerin^+^ DCs is regulated by RARα in a RA-concentration-dependent manner. RARα promotes the development of these DC populations in hypo-RA conditions. However, systemic concentrations of RA effectively inhibit the generation of these DC populations. Our results provide new insights into the development of LCs and langerin^+^ cDCs.

## Results

### LC development is defective in *Rara-*deficient mice

**Fig. 1:**
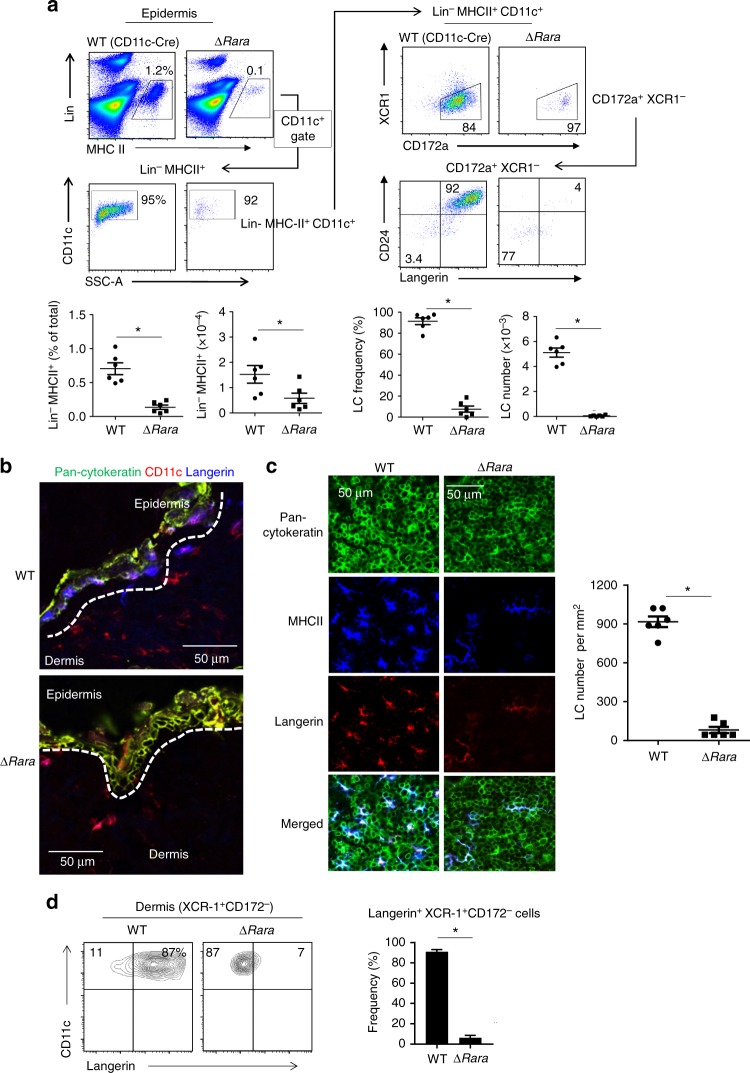
LC development requires RARα. **a** Frequency of langerin^+^ cells in the epidermis of WT and ∆*Rara*^CD11c^ mice. **b** Immunofluorescence detection of langerin^+^ DCs in cross-sections of the ear skin of WT and ∆*Rara*^CD11c^ mice. The white dashed lines indicate the border between the epidermis and dermis. **c** Immunofluorescence detection of langerin^+^ DCs in epidermal sheets of the ear skin of WT and ∆*Rara*^CD11c^ mice. Representative and combined data are shown. **d** Langerin expression by dermal DCs from WT vs. ∆*Rara*^CD11c^ mice. As WT control mice, C57BL/6 or CD11C-Cre-GFP mice were used. Representative and combined data (*n* = 4–8) from at least 3 experiments are shown. *Significant differences from control mice by Mann-Whitney U test (*p* < 0.05, unpaired, 2-sided). Error bars are defined as s.e.m

There have been efforts to develop LC-like cells in vitro from mouse BM cells^10,33^, but none were able to effectively induce langerin expression. In line with this, the generation of langerin^+^ cells was ineffective in a medium containing regular fetal bovine serum (FBS) (Supplementary Fig. 1a). We used a medium with charcoal-treated FBS, which contains reduced levels of RAs^34^, to induce LC differentiation with the two LC-promoting cytokines GM-CSF and TGF-β1. CD11c^+^ cells expressing the well-established LC markers, langerin and EpCAM, were readily generated in the charcoal-treated FBS medium (Supplementary Fig. 1b). The frequencies of langerin^+/-^EpCAM^+^ cells in the culture were highest on day 3. *Rara* mRNA is expressed by the BM-derived LC-like cells, and this expression was decreased by RA (Supplementary Fig. 2a). *Rara* expression was higher in CD11c^+^ cells cultured in the BM-LC than in a BM-DC condition. Moreover, it was highly expressed by primary LC cells from 3-day old mice (Supplementary Fig. 2a). This expression level was higher than those of epidermal CD11c^+^ MHC-II^+^ cells that had not yet expressed langerin (pre-LCs) from newborn mice and of dermal CD11c^+^ MHC-II^+^ and CD45-negative epidermal tissue cells from 3-day old mice (Supplementary Fig. 2b). Publicly available microarray data also indicate that LCs expressed *Rara* at a level higher than many DC populations in lymphoid tissues (Supplementary Fig. 2c, ImmGen). To determine the function of RARα in LC development, we created ∆*Rara*^CD11c^ (CD11c-Cre × floxed *Rara*) mice with the exon 3 of the *Rara* gene deleted specifically in CD11c^+^ cells (Supplementary Fig. 3). The frequency and numbers of CD11c^+^MHC-II^+^ cells were drastically decreased in the epidermis of ∆*Rara*^CD11c^ mice (Fig. 1a). Interestingly, langerin^+^ CD11c^+^MHC-II^+^ cells were almost absent in the epidermis of ∆*Rara*^CD11c^ mice (Fig. 1a, Supplementary Fig. 4). The few langerin^-^ CD11c^+^ cells, present in the epidermis of ∆*Rara*^CD11c^ mice, were abnormal in that they also lack CD24 expression (Fig. 1a, Supplementary Fig. 4). cDCs are largely divided into DC1 and DC2 in many tissues with high heterogeneity within each subset. Numbers of both XCR1^+^CD172^-^ DC1 and XCR1^-^CD172^+^ DC2 subsets were decreased in the dermis of ∆*Rara*^CD11c^ mic (Supplementary Fig. 5). Immunofluorescence microscopy confirmed that epidermal MHC-II^+^ cells (i.e., LCs) were largely absent in ∆*Rara*^CD11c^ mice with decreased numbers of dermal DCs (Fig. 1b). A few epidermal langerin^+^ cells, not readily detected by flow cytometry, were found in the epidermis of ∆*Rara*^CD11c^ mice. These cells had an abnormally elongated cell shape different from that of typical LCs (Fig. 1c). Significantly fewer langerin^+ ^DC1 cells were found in the dermis, skin-draining lymph nodes, lung and spleen of ∆*Rara*^CD11c^ mice compared to WT mice (Fig. 1d and Supplementary Fig. 6). Additionally, a moderate decrease in the number or frequency of the DC subsets was observed in the lung (Supplementary Fig. 7), indicating the possibility that RARα may also affect some non-LC conventional DC subsets. We focused this study on LCs and langerin^+^ cells, which were most clearly affected by RARα deficiency.

### Defective LC development in *Rara-*deficient progenitors

**Fig. 2:**
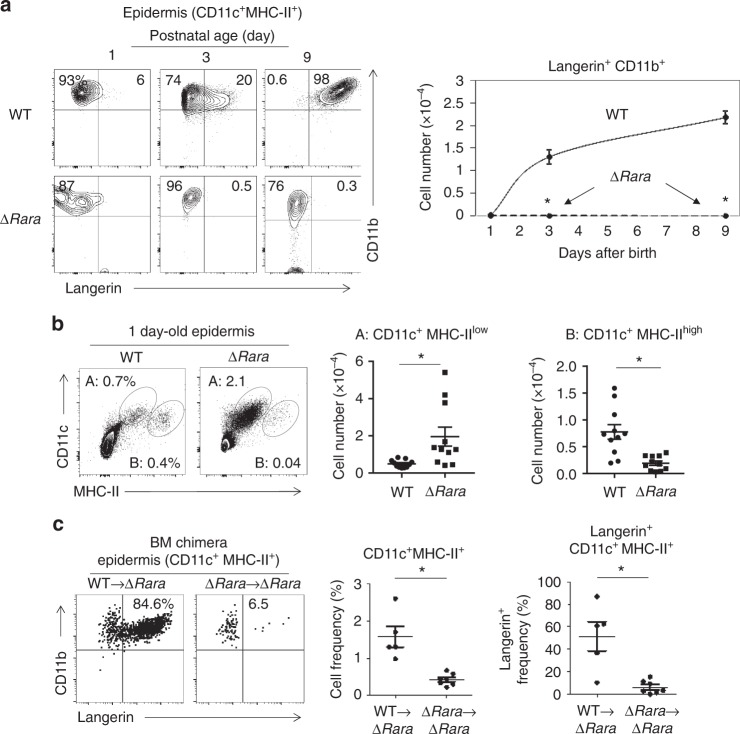
LC development in newborn mice and following BM transfer requires RARα. **a** Kinetics of langerin up-regulation on CD11c^+^ MHC-II^+^ cells in the epidermis of WT and ∆*Rara*^CD11c^ mice determined at postnatal day 1, 3, and 9. **b** Phenotype of epidermal DCs in new born (1-day old) mice. Trunk skin of WT and ∆*Rara*^CD11c^ mice was examined for CD11c^+^ MHC-II^+^ cells. **c** A BM reconstitution study from WT or ∆*Rara*^CD11c^ mice into lethally irradiated ∆*Rara*^CD11c^ mice. As WT control mice, we used either C57BL/6 or CD11C-Cre-GFP mice. The frequency of total CD11c^+^ MHC-II^+^ cells and epidermal langerin^+^ cells (% of CD11c^+^ MHC-II^+^) were examined 12–15 weeks post-BM transfer. Representative and combined data (*n* = 4–8) from at least 3 experiments are shown. *Significant differences from control mice by Mann–Whitney U test (*p* < 0.05, unpaired, 2-sided). Error bars are defined as s.e.m

Langerin up-regulation on CD11c^+^ MHC-II^+^ CD11b^+^ cells occurs right after birth in mice with massive proliferation of this cell population^35^. However, the early langerin up-regulation was aborted in ∆*Rara*^CD11c^ mice (Fig. 2a). In the epidermis of newborn ∆*Rara*^CD11c^ mice, the frequency and number of CD11c^+^ MHC-II^low^ cells were abnormally increased at the expense of CD11c^+^ MHC-II^high^ cells (Fig.2b, Supplementary Fig.8a). The CD11c^+^ MHC-II^low^ cells did not persist and soon disappeared on day 3 in the skin of ∆*Rara*^CD11c^ mice (Supplementary Fig.8a). The langerin^-^ CD11c^+^ MHC-II^high^ cells slightly increased in number in the skin of 9-day-old ∆*Rara*^CD11c^ mice but failed to expand to the level of langerin^+^ CD11c^+^ MHC-II^high^ cells (Supplementary Fig.8a). Ki-67 expression, which is strictly expressed by proliferating cells, was decreased on both the CD11c^+^ subsets from the ∆*Rara*^CD11c^ compared to WT newborn mice (Supplementary Fig.8b), which is in line with defective expansion and differentiation. Annexin V staining indicates that there were few dead cells in the CD11c^+^ MHC-II^low^ and MHC-II^high^ populations. While the importance is unclear, we observed somewhat lower cell death in the ∆*Rara*^CD11c^ CD11c^+^ MHC-II^low^ population, compared to their WT counterpart (Supplementary Fig.8b).

In postnatal life, LCs can be generated from BM-derived precursors. In order to determine if RARα is required for LC generation from BM-derived cells, we performed a BM reconstitution study from WT or ∆*Rara*^CD11c^ mice to ∆*Rara*^CD11c^ mice (Supplementary Fig.9a). While WT BM transfer into ∆*Rara*^CD11c^ mice generated langerin^+^ cells, BM transfer from ∆*Rara*^CD11c^ to ∆*Rara*^CD11c^ mice failed to generate them (Fig. 2c, Supplementary Fig.9b). BM transfer into WT mice did not lead to successful reconstitution with donor cells in the epidermis (Supplementary Fig.9a) as expected due to the known radio-resistance of recipient LCs in the skin^5^. Overall, these results show that ∆*Rara*^CD11c^ progenitors are defective in generating langerin^+^ CD11c^+^MHC-II^+^CD11b^+^ cells in the skin after BM transplantation.

### RA negatively regulates BM-LC generation **in vitro**

**Fig. 3:**
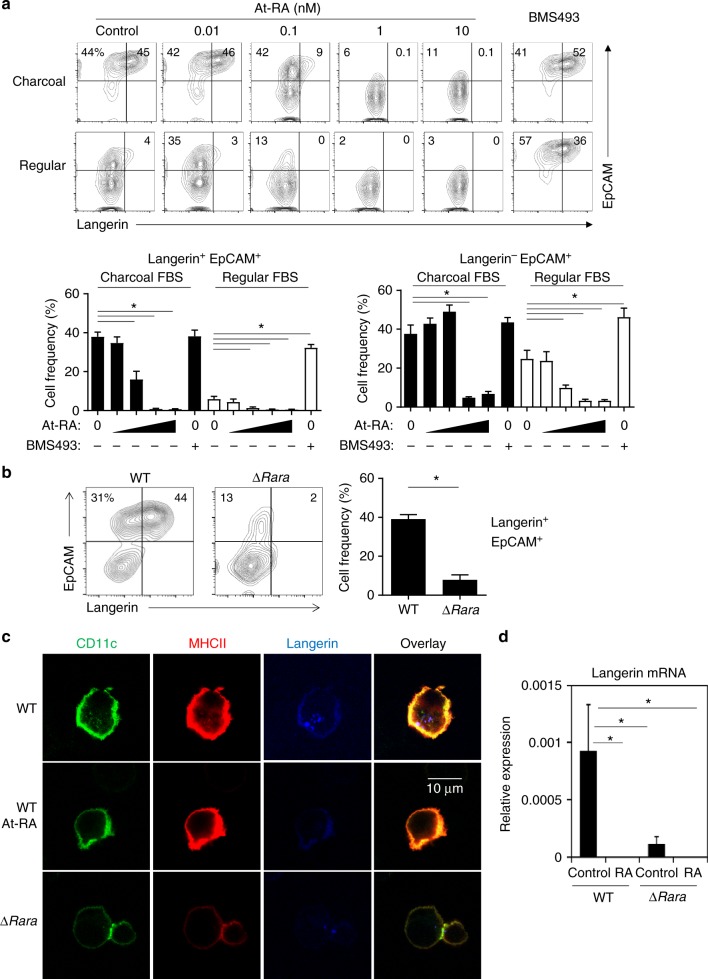
RA negatively regulates LC development. **a** RA suppresses langerin^+^ cell (BM-LC) generation in vitro, while the RAR antagonist BMS493 enhanced BM-LC generation. For BM-LC culture, BM cells were cultured in GM-CSF and TGFβ1 for 3 days in the presence of At-RA (0.01, 0.1, 1 and 10 nM) or BMS493 (100 nM) in media containing charcoal-treated or regular FBS. Frequencies of indicated CD11c^+^ cells are shown in graphs. **b** Defective BM-LC generation from BM cells of ∆*Rara*^CD11c^ mice. BM cells were cultured in a medium containing charcoal-treated FBS. **c** Confocal fluorescent microscopy of langerin expression by CD11c^+^ BM cells cultured in the LC-inducing condition without and with RA (1 nM). **d** Expression of *Rara* mRNA by CD11c^+^ BM cells cultured in the LC-induction condition without or with RA (1 nM). Normalized values for a housekeeping gene (GAPDH) are shown. Representative and combined data (*n* = 3–8) from at least 3 experiments are shown. *Significant differences from control or between indicated groups by one-way ANOVA with Bonferroni multiple comparisons (**a**) or Mann–Whitney U test (*p* < 0.05, unpaired, 2-sided, **b**, **d**). Error bars are defined as s.e.m

We, next, investigated the effects of RAR agonists and antagonists on generation of langerin^+^ cells in vitro. Interestingly, RA, when added to cultures of BM cells in the LC-inducing condition, effectively suppressed the up-regulation of EpCAM and langerin, whereas BMS493, which blocks the effect of RA by enhancing nuclear corepressor (NCoR) interaction with RARα ^36^, enhanced expression of the LC markers (Fig. 3a). Suppression was clear even at 0.1 nM of RA, and complete suppression was achieved even at 1 nM of RA. Unlike WT BM cells, only a few percent of ∆*Rara*^CD11c^ BM cells expressed langerin in charcoal-treated FBS (Fig. 3b). While the effects were not statistically significant (Supplementary Fig.10a–c), EpCAM up-regulation was detected on some ∆*Rara*^CD11c^ CD11c^+^ cells, and this expression was somewhat regulated by RA and BMS493 (Supplementary Fig.10a–c).

We further verified the regulation of langerin expression by a surface and intracellular co-staining approach. Both ∆*Rara* epidermal CD11c^+^ MHC-II^+^ cells and ∆*Rara* BM cells, cultured in the LC-induction condition, have defective surface and intracellular langerin expression (Supplementary Fig. 11a, b). This indicates that the defective langerin expression is not the result of simple internalization of langerin. Also, confocal imaging revealed that langerin protein expression was defective in both surface and intracellular compartments of ∆*Rara*^CD11c^ cells and was suppressed by RA (Fig. 3c). We observed that langerin expression in BM-LCs was suppressed at the mRNA level by RA, as well as by *Rara* deficiency (Fig. 3d). RA did not decrease existing langerin expression on and in primary LCs (Supplementary Fig. 11c).

Upon culture, LCs up-regulate the expression of CD40, CD86 and CCR7, but down-regulate E-cadherin (Supplementary Fig. 12a). During the culture, RA did not affect the change of these surface markers on primary LCs (Supplementary Fig. 12a). RA, when added during BM-LC differentiation, even suppressed the expression of CD40 and CD86 (Supplementary Fig. 12b). We also examined the possibility that RA at a physiological concentration influences the emigration of langerin^+/−^ CD11c^+^ MHC-II^+^ cells from WT and ∆*Rara* ear explants in response to a CCR7 ligand (CCL19). Both WT langerin^+^ and langerin^-^ CD11c^+^ MHC-II^+^ cells emigrated in response to CCL19, but RA had no effect on this migration (Supplementary Fig. 13). No migration of ∆*Rara* langerin^+^ CD11c^+^ MHC-II^+^ cells was detected which is probably due to their paucity in the skin. On the other hand, CCL19-dependent migration of ∆*Rara* langerin^-^ CD11c^+^ MHC-II^+^ cells was detected (Supplementary Fig. 13), albeit at low levels compared to WT cells, reflecting their smaller population size in the skin. These results indicate that physiological concentrations of RA or RARα deficiency are not likely to affect maturation or emigration of skin langerin-expressing cells.

Next, the impact of decreased RA levels on LCs was examined utilizing vitamin A deficient (VAD) mice. While the already high LC frequency in the epidermis was unchanged, the frequency of dermal langerin^+^ cells and their expression of langerin were significantly increased in adult VAD mice (Supplementary Fig. 14a–c). Thus, vitamin A metabolites negatively regulate the langerin^+^ dermal DCs.

### Impact of RARα deficiency and RA on gene expression

**Fig. 4:**
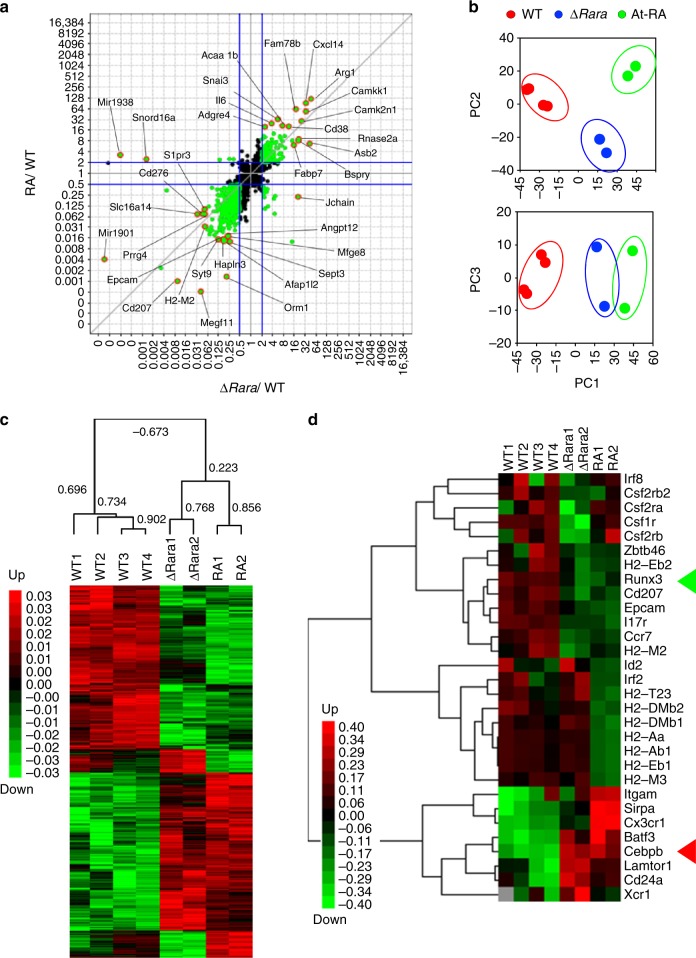
The effects of RARα deficiency vs. RA on gene expression in BM-derived DCs cultured in a LC-inducing condition. For all panels in this figure, RNA-seq was performed on WT and ∆*Rara*^CD11c^ BM cells cultured in charcoal-treated FBS medium containing GM-CSF and TGFβ1 for 3 days. WT cells were cultured with or without At-RA (10 nM). 2–4 independent samples were examined for each group. **a** Scatter plot analysis of RPKM (Reads Per Kilobase of transcript per Million mapped reads) values for ∆*Rara*^CD11c^ vs. WT for *X*-axis and RA vs. WT for *Y*-axis. 2619 differentially expressed genes were selected based on T-test (*P* < 0.2) adjusted with Benjamini–Hochberg procedure of the Multiplot Studio (Version 1.5.29, GenePattern). Total 8 samples were examined by RNA-seq (4 WT, 2 ∆*Rara*^CD11c^, and 2 At-RA). Genes highlighted in orange are the top 10 up- or down-regulated genes. **b** Principal component analysis (PCA) of the 2619 selected genes based on their RPKM values. **c** A Treeview showing hierarchical clustering of 2619 genes commonly or differentially regulated in BM-derived DCs cultured in a LC-inducing condition. Pearson correlation indices are shown. **d** A Treeview showing hierarchical clustering of manually selected genes from the differentially regulated 2619 genes. The green and red triangles respectively highlight *Runx3* and *Cebpb* genes. Error bars are defined as s.e.m

To gain insights into the functions of RARα and RA in regulating gene expression during LC differentiation, we performed an RNA-seq study on cultured BM cells from WT and ∆*Rara*^CD11c^ mice in the LC-inducing condition (Fig. 4). Multiplot, hierarchical clustering and principal component analyses (PCA) identified a high level of similarity between the effects of RA and RARα deficiency, increasing or decreasing largely overlapping groups of genes (Fig. 4a–c). Genes associated with LC or DC development, such as *Runx3* and *Cebpb*, were co-regulated by RARα deficiency and RA (Fig. 4d). Also, the expression of *Epcam1, Ccr7*, and *Cd207* were co-suppressed by RA and RARα deficiency. The expression of *Itg-αM, Sirpa* and *Cx*_*3*_*cr1* was increased by RA, whereas the expression of *Lamptor1* and *Xcr1* was increased by RARα deficiency (Fig. 4d). Additionally, top 50 up- and down-regulated by RA and RARα deficiency, including *Snord88a* (Small Nucleolar RNA SNORD88), *Cd276* (B7-H3), and *Mfge8* (Milk fat globule-EGF factor 8 protein), are listed in Supplementary Fig. 15a and b. These results indicate that RARα deficiency exerts a powerful influence on gene expression during LC-like cell differentiation. The RNA-seq analysis revealed that the effects of RARα deficiency and RA treatments have largely similar negative effects on BM-LC development.

### Runx3 and C/EBPβ are reciprocally regulated by RARα and RA

**Fig. 5:**
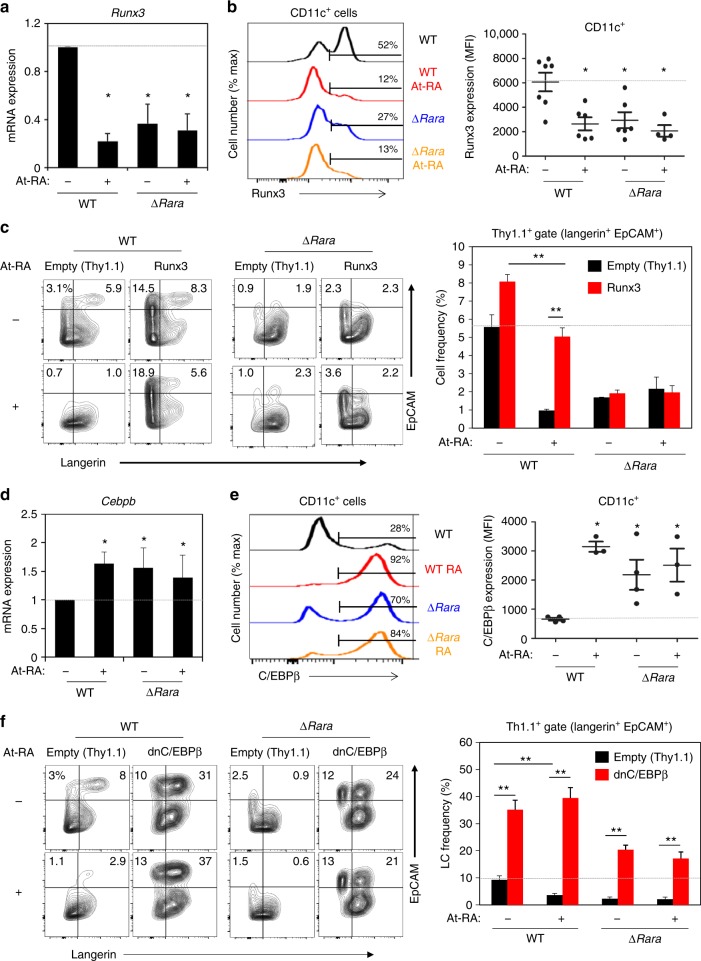
RA and RARα reciprocally regulate the expression of the positive and negative LC-regulating transcription factors, Runx3 and C/EBPβ. **a** Impact of RA and RARα deficiency on Runx3 expression at mRNA level. **b** Impact of RA and RARα deficiency on Runx3 expression at protein level. **c** Effect of enforced Runx3 expression on BM-derived LC differentiation in the presence and absence of RA. The data shown are gated for transduced Thy1.1^+^CD11c^+^ cells. **d** Impact of RA and RARα deficiency on expression of *Cebpb* mRNA. **e** Impact of RA and RARα deficiency on expression of C/EBPβ protein. **f** Effect of dnC/EBPβ on BM-derived LC differentiation in the presence and absence of RA. BM cells from WT or ∆*Rara*^CD11c^ mice were cultured with GM-CSF and TGFβ1 for 5 days (3 days following retroviral transduction) in the presence of At-RA (10 nM except in panels **c** and **f** where 0.1 nM was used) in a medium containing charcoal-treated FBS. Representative and combined data (*n* = 3–7) from at least 3 experiments are shown. Significant differences from controls by one-way ANOVA with Bonferroni adjustments (*p* < 0.05)* or between indicated groups by two-way ANOVA with Tukey adjustments (*p* < 0.05)**. Error bars are defined as s.e.m

Runx3, a transcription factor that promotes LC generation^14^, was one of the genes down-regulated by both RA and RARα deficiency. We confirmed this down-regulation at mRNA and protein levels (Fig. 5a, b). Down-regulation of Runx3 provides a potential mechanism for the decreased LC generation by RA. We over-expressed Runx3 with a retroviral gene transfer method and examined if this abolished the inhibitory effect of RA and RARα deficiency (Fig. 5c). The enforced Runx3 expression abolished the suppressive effect of RA. However, the enforced Runx3 expression did not normalize the LC generation defect of ∆*Rara*^CD11c^ cells, indicating that the effects of RA and RARα deficiency on LC development are not identical.

*Cebpb*, a gene that encodes a transcription factor that suppresses LC but promotes macrophage differentiation^18,37^, was up-regulated by RA and in RARα deficiency. C/EBPβ up-regulation was confirmed at mRNA and protein levels (Fig. 5d, e). To assess the role of up-regulated C/EBPβ by RA and RARα deficiency in LC-like cell development, we suppressed C/EBPβ activity by enforced expression of dominant negative (dn) C/EBPβ with a retroviral approach. Hyper-induction of WT LCs by dnC/EBPβ indicates C/EBPβ’s powerful suppressive effect on LC generation (Fig. 5f). Importantly, enforced expression of dnC/EBPβ abolished not only the suppressive effect of RA but also that of RARα deficiency (Fig. 5f). Thus, C/EBPβ regulation by the RA-RARα axis is functionally important for LC development.

### DNA-binding is important for the function of RARα

**Fig. 6:**
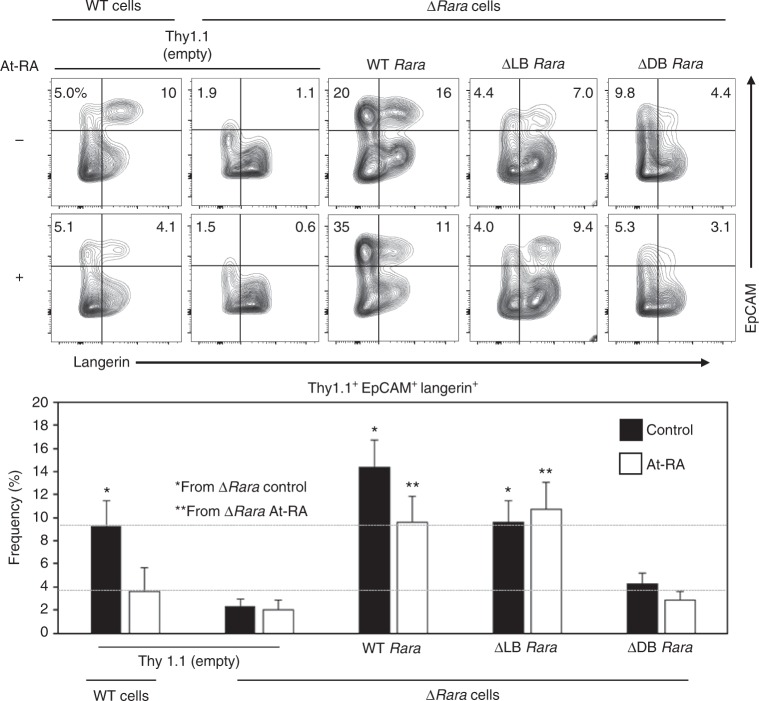
The DNA binding, but not ligand binding, function of RARα is required for BM-LC generation. Impact of RARα variants on BM-derived LC differentiation in vitro. BM cells were transduced with retroviral vectors harboring WT and variant RARα genes that lack ligand binding (ΔLB) or DNA binding (ΔDB) activity and differentiated with GM-CSF and TGFβ1 for 5 days in the presence of RA in medium containing charcoal-treated FBS. The data shown are for transduced Thy1.1^+^CD11c^+^ cells. Representative and combined data are shown. At-RA was used at 0.1 nM. Representative and combined data from 5 experiments are shown. *^,^**Significant differences by two-way ANOVA with Tukey adjustments (*p* < 0.05). Error bars are defined as s.e.m

To gain further mechanistic insights into the RARα function in regulating LC differentiation, we expressed genes for WT and mutant forms of RARα in ∆*Rara*^CD11c^ cells. Retroviral transduction of wild type *Rara* restored the LC differentiation defect in ∆*Rara*^CD11c^ cells. Interestingly, the *Rara* G303E mutant gene with a defective ligand binding domain (*Rara-*ΔLB) was able to rescue the differentiation defect (Fig. 6). However, the *Rara* C105G mutant gene with a defective DNA binding domain (*Rara-*ΔDB) could not rescue the defect, underlining the importance of DNA binding capacity of RARα in regulating LC development. Thus, the positive function of RARα is mediated through DNA binding.

### The RARα-RA axis also regulates human LC differentiation

**Fig. 7:**
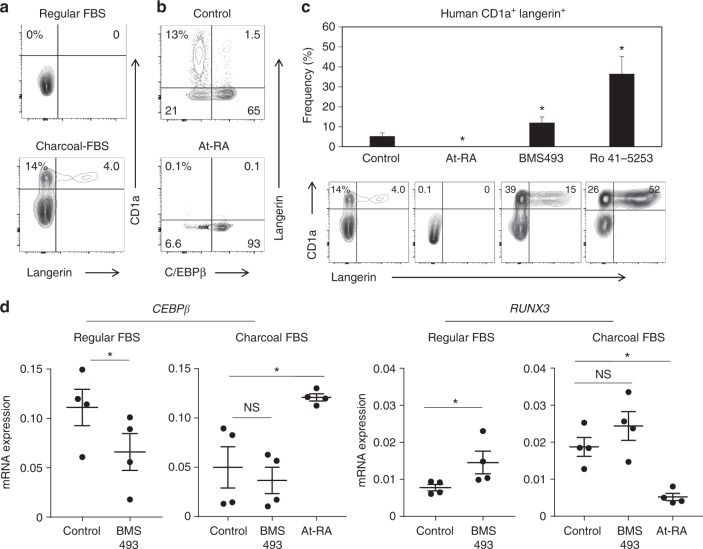
RA regulates *CEBPb* and *RUNX3* expression and human langerin^+^ cell differentiation from blood monocytes. **a** Human LC differentiation from blood monocytes in regular vs. charcoal FBS. **b** At-RA promotes C/EBPβ^+^ non-LC differentiation. **c** RA and RARα antagonists reciprocally regulate human LC differentiation. **d** Effects of At-RA and BMS493 on the expression of human *CEBPb* and *RUNX3*. Human blood CD14^+^ monocytes were cultured in GM-CSF and TGF-β1 for 5–7 days in media containing charcoal FBS except in panel A where regular FBS was also used. At-RA was used at 1 nM, and BMS493 and Ro4153 were used at 500 nM. Representative and combined data (*n* = 5–7) from at least 5 experiments are shown. *Significant differences from control by Mann–Whitney U test (*p* < 0.05, unpaired, 2-sided). Error bars are defined as s.e.m. Langerhans cells (LC) and langerin-expressing conventional dendritic cells are made from distinct progenitors and enriched in the distinct microenvironments of the skin. Here the authors show that these immune cells are regulated by RARα via simultaneous induction of LC-promoting Runx3 and repression of LC-inhibiting C/EBPβ

Finally, we studied whether RA also regulates human LC differentiation. Langerin^+^ LC-like cells can be differentiated from peripheral blood CD14^+^ monocytes or CD1c^+^ DCs^17,38^. As reported by others, normal fetal bovine serum (FBS) does not support langerin^+^ LC-like cell generation (Fig. 7a). However, we found that charcoal-treated FBS was conducive for LC-like cell generation from CD14^+^ monocytes. This indicates that there is a factor (or factors) in blood that inhibits human LC differentiation. The results with mouse LC cells strongly suggest that RA is such a factor that restrains LC differentiation. Indeed, RA, when added to culture even at a low concentration (1 nM), completely suppressed the generation of langerin^+^ CD1a^+^ LC cells (Fig. 7b). Considering the significant concentrations (~5 nM) of RA in human blood^39^, our results indicate that physiological levels of RA have the potential to effectively suppress LC differentiation in systemic tissues. Two different RARα antagonists, BMS493 and Ro41-5253, enhanced human LC-like cell generation (Fig. 7c), which suggests that RA in regular FBS is indeed a factor that suppresses LC generation. Langerin^+^ cells were mostly C/EBPβ^−^ cells, and the generation of these cells was effectively suppressed by RA (Fig. 7b). At mRNA level, *CEBPb* expression was decreased by BMS493 in the regular-FBS medium and increased by RA in the charcoal-treated FBS medium (Fig. 7d). The pattern of RUNX3 expression was exactly the opposite, increased by BMS493 and decreased by At-RA in the human LC-like cells. Overall, RARα ligands and antagonists reciprocally regulate LC-like cell differentiation from human blood monocytes.

## Discussion

In this study, we demonstrated that a regulatory pathway shaped by RARα plays a crucial role in the development of LCs and langerin-expressing DCs. We demonstrated that RARα is required for LC differentiation in vitro and in vivo. Interestingly, RARα drives LC differentiation and generation of langerin-expressing DCs in hypo-RA conditions. In systemic concentrations, RA generates a negative signal to effectively suppress LC differentiation. Thus, RARα plays a highly sophisticated role in the development of LCs and langerin^+^ cDCs in a RA-concentration-dependent manner.

LCs are developed from both embryonic progenitors and bone marrow-derived cells. We demonstrated that RARα-deficient progenitors fail to give rise to LCs and langerin^+^ cDCs in the skin. We speculate that the CD11c^+^ MHC-II^dim^ cells in the epidermis of newborn mice could be the precursors unable to develop into LCs in *Rara* deficiency. Langerin expression on DCs in non-skin tissues was also decreased in RARα-deficient mice. We also demonstrated that RARα-deficient CD11c^+^ BM progenitors fail to differentiate to LCs in the skin during adult life. Therefore, RARα is required for LC development from both fetal and BM origins in newborn and adult mice respectively.

A question is how RARα regulates LC development. RARα is a transcription factor that functions through both DNA binding and non-binding mechanisms^40^. A major way to trigger RARα activation is through ligand binding, which induces conformational changes to recruit co-activators and dissociate co-repressors. This can cause epigenetic changes and regulates gene expression. However, our results regarding the regulation of LC development indicate that RARα has a clear positive function in hypo-ligand conditions. This phenomenon is supported by the fact that RARα can epigenetically regulate gene expression in a manner independent of retinoid ligands as documented previously^41^. It seems that RARα shapes the gene expression program conducive for LC generation. As we demonstrated, however, the function of RARα in regulating LC differentiation changes depending on the presence of its ligands (i.e., RAs).

While RARα is required for LC development, this appears to occur in hypo-RA conditions. The in vitro culture systems that were used in this study with charcoal-treated vs. regular FBS were useful to determine the negative role of RA in suppressing mouse BM-LCs. Charcoal depletes hydrophobic components, such as RAs, from FBS. Thus, the medium with charcoal-treated FBS represents a hypo-RA condition. The medium with regular FBS represents sub-systemic RA levels because it contains only 10% of FBS. While the charcoal-treated FBS condition allows BM-LC differentiation induced by the two cytokines, GM-CSF and TGF-β1, the differentiation did not readily occur in the regular FBS condition. Furthermore, exogenously added RA to the charcoal-treated FBS medium suppressed the generation of BM-LCs in a RA concentration-dependent manner. On the other hand, two different types of RARα antagonists: Ro41-5253 (a competitive inhibitor) and BMS493 (an inverse antagonist, blocking the effect of residual RA in the charcoal-treated FBS medium), increased the generation of the in vitro generated LCs. This indicates that the inhibitory function of RA is mediated through RARα. It is plausible in developing LCs that RA activation of RARα alters its ability to bind and recruit factors that affect gene expression and cell differentiation. This seemingly complex molecular regulatory networks require further studies to delineate.

We demonstrated that the DNA-binding ability of RARα is important for its regulation of BM-LCs. The DNA binding function of RARα can be mediated by several different ways, including direct binding to canonical retinoid acid response element (RARE) sequences, half RARE sites, and other incompletely identified DNA sequences. RARα can also bind DNA via tethering through protein-protein interaction with other proteins such as BZLF1 to exert their regulatory effects^42^. Indeed, many of the genes regulated by RA lack canonical RAREs^43^. Therefore, it is likely that the function of RARα in regulating LCs is the result of both direct and indirect or primary and secondary regulation of gene expression by the RARα-RA axis. The RA effect on LC differentiation could be mediated also by non-genomic functions of RA. For example, it has been reported that RA rapidly activates several kinases including p38 mitogen-activated protein kinase (MAPK) and p42/p44 extracellular signal-regulated kinases (Erk)^44,45^.

RARα-deficient BM progenitors, while they can’t effectively express langerin, can still express EpCAM albeit at reduced levels compared to WT BM progenitors. This indicates differential dependence of the expression of LC-associated molecules on RARα. Alternatively, some cells, which may have escaped the CD11c-Cre-induced *Rara* gene deletion or expressed the molecules before *Rara* gene deletion, have the potential to express the LC-associated molecules. Alternatively, major RA, such as At-RA and 9-cis-RA, work through additional retinoid receptors such as RARβ and RARγ isotypes. Moreover, RAs activate other receptors such as PPARβ/δ, RORβ, COUP-TFII and TR2/4^46^, which may also explain the difference between the effects of RARα and RA. This possibility, however, is not high because the affinity of RA to these receptors is significantly lower than that to RARα ^46^. Moreover, functional expression of these receptors should be esablished in LCs and langerin-expressing DCs.

Our results indicate that RARα and RA can regulate a number of genes in LC precursor cells. Indeed, we found that the *Cebpb* gene expression is up-regulated in RARα deficiency, and it is also increased by RA, effectively suppressing LC differentiation. The *Cebpb* gene encodes C/EBPβ, a transcription factor required for the development of certain macrophage subsets but suppressive of LC differentiation^18,37,47^. On the other hand, the expression of the pro-LC transcription factor Runx3 is suppressed in RARα deficiency. Therefore, these two genes are potential mediators of the RARα function to regulate LC differentiation. While some genes are direct targets of RARα, many genes are regulated indirectly. Further studies in this regard are necessary to delineate the regulatory mechanism in the reciprocal expression of the two transcription factors. Our RNA-seq study revealed that many more genes in addition to the two genes are regulated by RARα. The genes for EpCAM, CD207 (langerin), and MHC II molecules expressed by LCs were suppressed in RARα deficiency. Genes upregulated in RARα deficiency include macrophage-associated genes such as Arg1, CD38, IL-6, CXCL14, and Snai3. Interestingly, RA regulates the expression of these genes in a manner highly similar to RARα deficiency. We reason that the similar effects of RARα deficiency and RA on the global gene expression in developing LCs is probably due to the shared suppressive effect by these two conditions.

The RA-RARα axis also regulates human LC development, which was demonstrated in this study in an in vitro setting. It has been reported that serum (i.e., blood) contains fat-soluble factors that potently suppress human LC differentiation^48^ but such a suppressive factor has not been identified to date. Our results identify that RA is such a suppressive factor. Serum concentration of RA is ~5 nM. In general, 0.1–10 nM is a physiologically relevant concentration range of RA, which was effective in suppressing LC-like cell generation in vitro. As exemplified well in developing embryos^49^, RA levels in tissues are likely to be finely regulated by RA-producing and degrading cells. The epidermis-specific tissue tropism of LCs in the body together with the fairly high RA concentrations in the blood suggest that the RARα has the potential to effectively promote LC differentiation from precursors in the skin epidermis but probably suppress ectopic LC differentiation in relatively high-RA environments, such as blood circulation, lymphoid tissues, and gut tissues^50^. In this regard, the topological correlation between RA activity and LC development in the body should be established in the future. In sum, our findings raise the possibility that the development of LCs and LC-like cells is regulated by an environmental cue shaped by the levels of vitamin A metabolites and RARα function in precursors cells.

## Methods

### Animals, vitamin A deficiency, and topical retinoid treatment

All animal experiments were approved by the Animal Care and Use Committees at University of Michigan and Purdue University. CD11c-Cre GFP mice (stock # 007567) were purchased from the Jackson Laboratory (Bar Harbor, ME) and mated with RARα (f/f) mice^51^ to generate ∆*Rara*^CD11c^ mice. As WT control mice, we used either C57BL/6, RARα (f/f), and CD11C-Cre-GFP (including littermate and non-littermate) mice, all of which were not different from each other in LC development. The data from male and female mice were indistinguishable and combined. Vitamin A-deficient (VAD) and vitamin A-normal (VAN) mice were generated by feeding late-term (15–16 post coitus-day) pregnant females with AIN-93G-based custom diets containing retinyl acetate at 2500 IU/kg (VAN, TD. 07267, Harlan Teklad, Indianapolis, IN) or 0 IU/kg of diet (VAD, TD. 00158). Weaned mice were kept on the same diet for at least 10 weeks before examination of DC subsets. No randomization or blinding was performed for animal experiments.

### BM reconstitution study

BM cells (5 × 10^5^ cells per mouse) from WT or ∆*Rara*^CD11c^ mice were transferred i.v. into lethally irradiated (1,100 rads) ∆*Rara*^CD11c^ mice. Mice were examined 12–15 weeks after bone marrow transplantation.

### Cell isolation

Mouse ears were separated into two skin halves and treated with 0.5% trypsin (Alfa Aeser, Ward Hill, MA) at 37 °C for 30 min. Epidermis and dermis were separated and dermis was digested with collagenase IV (1–2 mg ml^−1^, Worthington, Lakewood, NJ). Epidermal cells were released after trypsin digestion without using collagenase treatment. The trunk skin of neonatal mice was similarly processed to obtain single cell suspensions. For qRT-PCR analysis of *Rara* and *Cd207* genes, CD11c^+^ cells from BM culture or CD11c^+^ cells and CD45-negative tissue cells from single suspensions of epidermis or dermis were isolated by magnetic selection with biotin-labeled CD11c (clone N418) and Mojosort^TM^ streptavidin nanobeads (BioLegend; purity > 90%). Gradient centrifugation of epidermal and dermal cells released by collagenase digestion was performed on a 40/70% Percoll gradient to enrich leukocytes.

### Induction of mouse BM-derived LC-like cells

For most experiments, BM cells were cultured for 3 days in complete RPMI-1640 medium supplemented with regular or charcoal-treated FBS (10%, Thermo-Fisher). GM-CSF (20 ng ml^−1^) and hTGF-β1 (10 ng ml^−1^) were added to generate LC-like cells. All cytokines were from BioLegend. When indicated, retinoids such as At-RA (1–10 nM) and BMS493 (100 nM) were added to culture. RA was dissolved in dimethyl sulfoxide (DMSO) and therefore DMSO at 0.1 nM was also used as a control.

### Retroviral expression of *Runx3, Cebpb*, and *Rara* genes in LCs

Mouse dn*Cebpb* (a gift from C. Vinson, Addgene plasmid #33363) and human *Rara* genes were sub-cloned into MSCV-Thy1.1 vector. Runx3-expressing MSCV-Thy1.1 has been described previously^52^. ΔLB(G303E) and ΔDB (C105G)-*Rara* mutant genes were cloned from pCMX-Epi-RARa-G303E, and RARa-C105G^53^ into MSCV-Thy1.1. 70–80% confluent Platinum-E retroviral packaging cells on a 10 cm-plate were transfected with retroviral vectors (10 μg) and pCL-Eco retrovirus packaging vector (4 μg) using Lipofectamine 2000 (Invitrogen). Culture supernatant from day 2 to 3 was collected and used to spin-infect BM cells (2.5 × 10^5^ per well) in the presence of polybrene (Sigma-Aldrich, 2 μg ml^−1^). Infected BM cells were cultured in a RPMI-1640 medium containing charcoal-treated FBS (10%), GM-CSF (20 ng/ml) and hTGF-β1 (10 ng ml^−1^) for 5 days with or without At-RA. Surface and intracellular molecules expressed by transduced Thy1.1^+^ CD11c^+^ cells were determined by flow cytometry.

### Induction of human monocyte-derived LC-like cells

Human CD14^+^ monocytes were sorted from peripheral blood cells. Histopaque-1077 (Sigma-Aldrich) was used to separate mononuclear cells from red blood cells. CD14^+^ cells were sorted with biotin-labeled anti-CD14 antibody (M5E2) and anti-biotin beads (Miltenyi Biotec, Auburn CA). CD14^+^ cells (95–97% pure) were cultured for 5–7 days in complete RPMI-1640 medium supplemented with regular or charcoal-treated FBS (10%, Thermo-Fisher). GM-CSF (20 ng ml^−1^) and hTGF-β1 (10 ng ml^−1^) were added along with retinoids such as At-RA and Ro41-5253. Cultured cells were stained with antibodies and examined by flow cytometry as described below or examined by qRT-PCR.

### Flow cytometry

Mouse cells were stained with antibodies to CD11c (clone N418), MHC-II (clone M5/114.15.2), EpCAM (clone G8.8), langerin (clone 4C7 and L31), CD11b (clone M1/70), C/Ebpβ (clone E299, Abcam, Cambridge, MA), Runx3 (clone 527327, R&D, Minneapolis, MN), CD24 (clone M1/69), CD26 (clone H194-112), CD172a (clone P84), and XCR-1 (clone ZET). Mouse DC subsets were stained as described by others with some modifications^54^. For human cells, antibodies to human CD14 (M5E2), CD1a (HI149), CD11c (BD Biosciences, B-ly6), C/EBPb (Abcam, E299), and CD207 (10E2) were used. Lineage-specific antibodies included CD3 (clone 145-2C11), NK1.1 (clone PK136), CD19 (clone 6D5), B220 (clone RA3-6B2), and CD64 (clone X54-5/7.1). When indicated, antibodies to CD45.2 (104), CD45.1 (A20), Ki-67 (16A8), CCR7 (4B12), CD40 (3/23), and CD86 (GL-1) were also used. Antibodies were purchased from BioLegend (San Diego, CA) unless indicated otherwise. For intracellular staining of transcription factors, cells were first stained for surface antigens, fixed and permeabilized using Fix/Perm buffer (Tonbo Bioscience, San Diego, CA) and then stained with antibodies to transcription factors in Perm buffer (Tonbo Bioscience). Cells were acquired by Canto II (BD Biosciences) or NovoCyte 3000 (ACEA Biosciences Inc.). For langerin intracellular staining, cells were first stained with antibodies to surface antigens, including langerin (clone 4C7, allophycocyanin-labeled, BioLegend), followed by fixation and permeabilization using the Fix/Perm buffer (Tonbo Biosciences Transcription Factor kit). Cells were then stained for intracellular langerin using a PE-conjugated antibody (clone L31, eBioscience). Annexin V staining was performed in a special binding buffer purchased from BioLegend.

### Skin DC emigration from ear explants

Ears from WT and ∆*Rara*^CD11c^ mice were split into dorsal and ventral halves and floated split side down on 1 ml RPMI medium containing 10% charcoal-treated FBS. CCL19 (500 ng ml^−1^) and RA (10 nM) were added as indicated at the beginning of the cultures. Because CCL19-dependent LCs emigration occurs mainly from 24 h, the cells were pre-incubated for 24 h and then the cells that emigrated during the next 24 h period in fresh medium were collected, stained with the Ghost cell viability dye (Tonbo Biosciences) and fluorescent antibodies to CD11c, MHC-II, EpCAM and langerin, and then counted with a NovoCyte 3000.

### Skin LC and BM-LC imaging

Epidermal sheets were separated from dermis after incubating in 0.5 M ammonium thiocyanate solution (Sigma Aldrich, St. Louis, MO) for 40 min at 37 °C. For frozen sections, ear skin was frozen in freezing medium (Sakura) and cryosections (8 μM) were made using a cryostat (Leica). Epidermal sheets and cryosections were fixed in acetone and stained with antibodies to pan-cytokeratin (AE1/AE3), MHC-II (M5/114.15.2) and langerin (4C7). Antibodies were from eBioscience or BioLegend. Images were obtained with a Leica SP5 II confocal system. For langerin expression in BM-LCs, cultured cells were stained for surface CD11c and MHC-II, fixed in 1% paraformaldehyde, and permeabilized with a saponin buffer for whole-cell staining of langerin. The stained cells were plated on Cell-Tak coated slide glasses and imaged with a Leica SP5 II confocal system.

### RNA-seq and bioinformatics

CD11c^+^ cells from BM culture were isolated by magnetic selection with biotin-CD11c (clone N418) and Mojosort^TM^ streptavidin nanobeads (BioLegend; purity > 90%). Total RNA was extracted using RNeasy Mini Kit (QIAGEN, Germantown, MD) and submitted to Purdue University Genomic Core Facility for sequencing. The samples were processed with the Illumina TruSeq Standed mRNA Sample Prep Kit (Illumina Inc. San Diego, CA). Following quantification with KAPA Library Quantification Kit (KAPA Biosystems, Wilmington, MA)_,_ the libraries were sequenced with a HiSeq 2500 using v3 chemistry to generate paired-end 100 base reads. Sequencing data were analyzed using FastQC (Babraham Bioinformatics, Cambridge, UK) for quality control and mapped to the mouse genome (UCSC mm10) using STAR RNA-seq aligner. Reads distribution across the genome was assessed using bamutils. Uniquely mapped sequencing reads were assigned to mm10 refGene genes using featureCounts with the following parameters: “-s 2 –p –Q 10”. Quality control of sequencing and mapping results was summarized using MultiQC. Genes with read count per million (CPM) < 0.5 in more than 2 of the samples were removed. The data were normalized using the trimmed mean of M (TMM) values method. Differential expression analysis was performed using edgeR. *T*-test *P*-value adjusted by Benjamini–Hochberg procedure was computed using Multiplot Studio (GenePattern, Broad Institute) to select a set of genes for further analysis. Multiplot Studio, hierarchical clustering, and TreeView from GenePattern were used to visualize differentially expressed genes. Principal component analysis (PCA) was performed using the WebMeV of TM4 applications (mev.tm4.org). Gene set enrichment analysis (GSEA, Broad Institute) was used to generate heat maps of top 50 up- or down-regulated genes.

### Quantitative real-time PCR

Total RNA was extracted with TRIzol and cDNA synthesis was performed with the High-Capacity cDNA Reverse Transcription Kit (Thermo Scientific, Grand Island, NY). Quantitative real-time PCR was performed with Maxima® SYBR Green/ROX qPCR Master Mix (Thermo Scientific) using the primers described in Supplementary Table 1. All data are shown as relative expression levels following normalization with GAPDH expression.

### Statistics

Sample sizes (6–11), particularly for animal experiments, were chosen by 2-sided power analysis based on at least 50% difference in population values, standard deviation of 30–40%, α value of 0.05, and desired power of 0.80. In performing flow cytometry or qRT-PCR, failed cell isolation based on low cell yields ( < 10% of average yields) or purity ( < 85%) were excluded. Normality and variance were not intentionally examined because sample size is too small (3–8) to have sufficient power. Statistical significance of differences between two groups were obtained mostly by Mann–Whitney U test (*p* < 0.05, unpaired, 2-sided). Complex data were examined by one-way ANOVA with Bonferroni’s correction or two-way ANOVA with Tukey’s multiple comparison test. Error bars in all figures indicate SEM. All experiments were performed at least 3 times for reproducibility.

## Electronic supplementary material

Supplementary Information

## Data Availability

The BM-LC RNA sequencing data have been deposited in Gene Expression Omnibus under the accession code (GSE101991).
