# Supplementary material for: RARα supports the development of Langerhans cells and langerin-expressing conventional dendritic cells
Source: Nat Commun. 2018 Sep 25;9:3896. doi: 10.1038/s41467-018-06341-8 (PMC6156335; doi:10.1038/s41467-018-06341-8)
Supplement: Supplementary file 1 — Supplementary Information [file 41467_2018_6341_MOESM1_ESM.pdf]

Supplementary Information

RARα supports the development of Langerhans cells and langerin-expressing conventional dendritic cells

Seika Hashimoto-Hill, Leon Friesen, Sungtae Park, Suji Im, Mark H. Kaplan, and Chang H. Kim

Address Correspondence to Chang Kim, chhkim@med.umich.edu

Supplementary Fig. 1 to Fig. 15

Supplementary Table 1

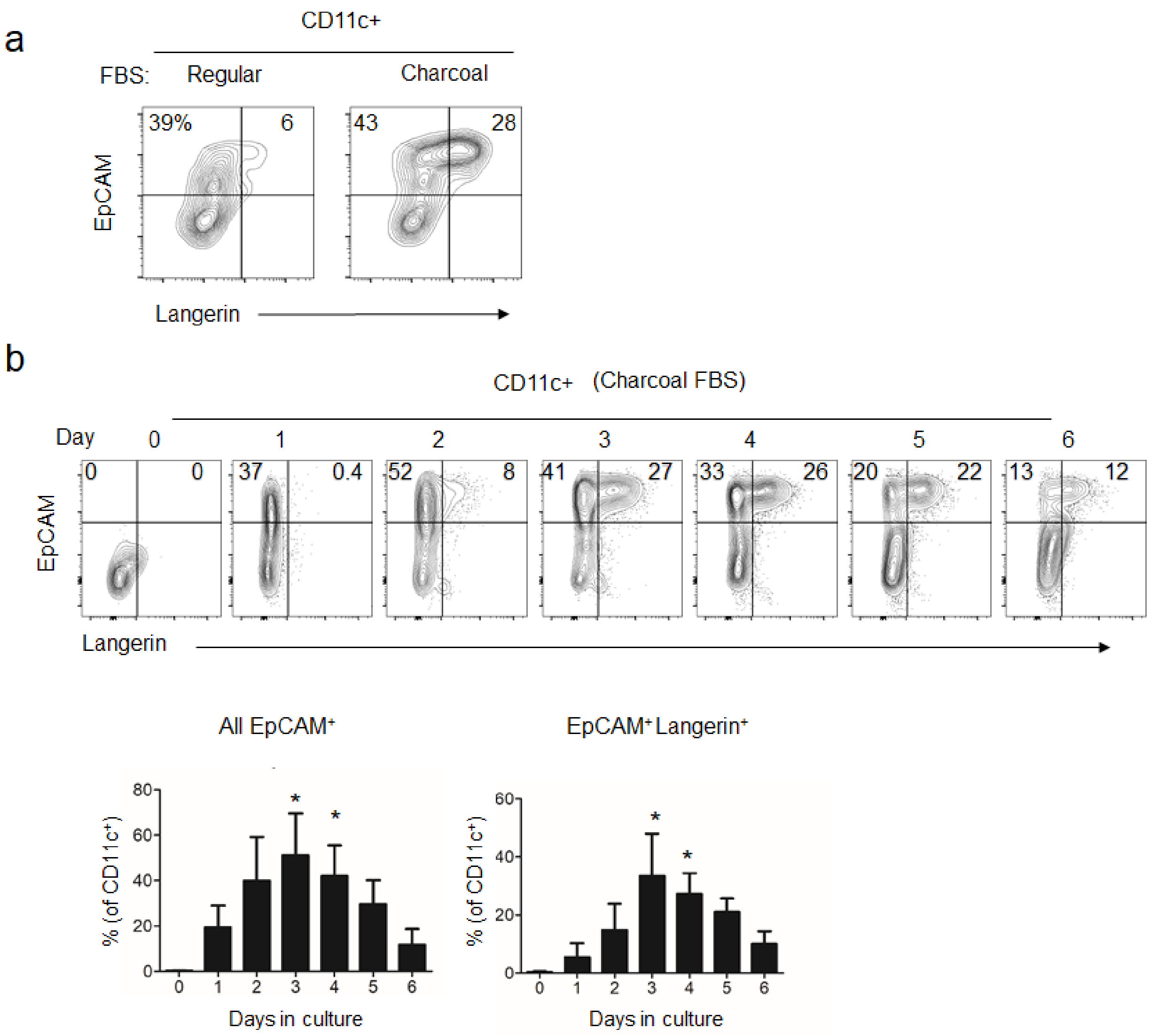

Supplementary Fig. 1. Induction of BM-derived langerin^{+} cells (BM-LC) *in vitro*. BM cells were cultured for 3 days (a) or indicated time periods (b) in complete RPMI-1640 medium supplemented with regular or charcoal-treated FBS (10%, Thermo-Fisher), GM-CSF (20 ng/ml) and hTGF-β1 (10 ng/ml). *Significant differences from day 0 ($p<0.05$; $n=3$) by Mann-Whitney U test ($p<0.05$, unpaired, 2-sided).

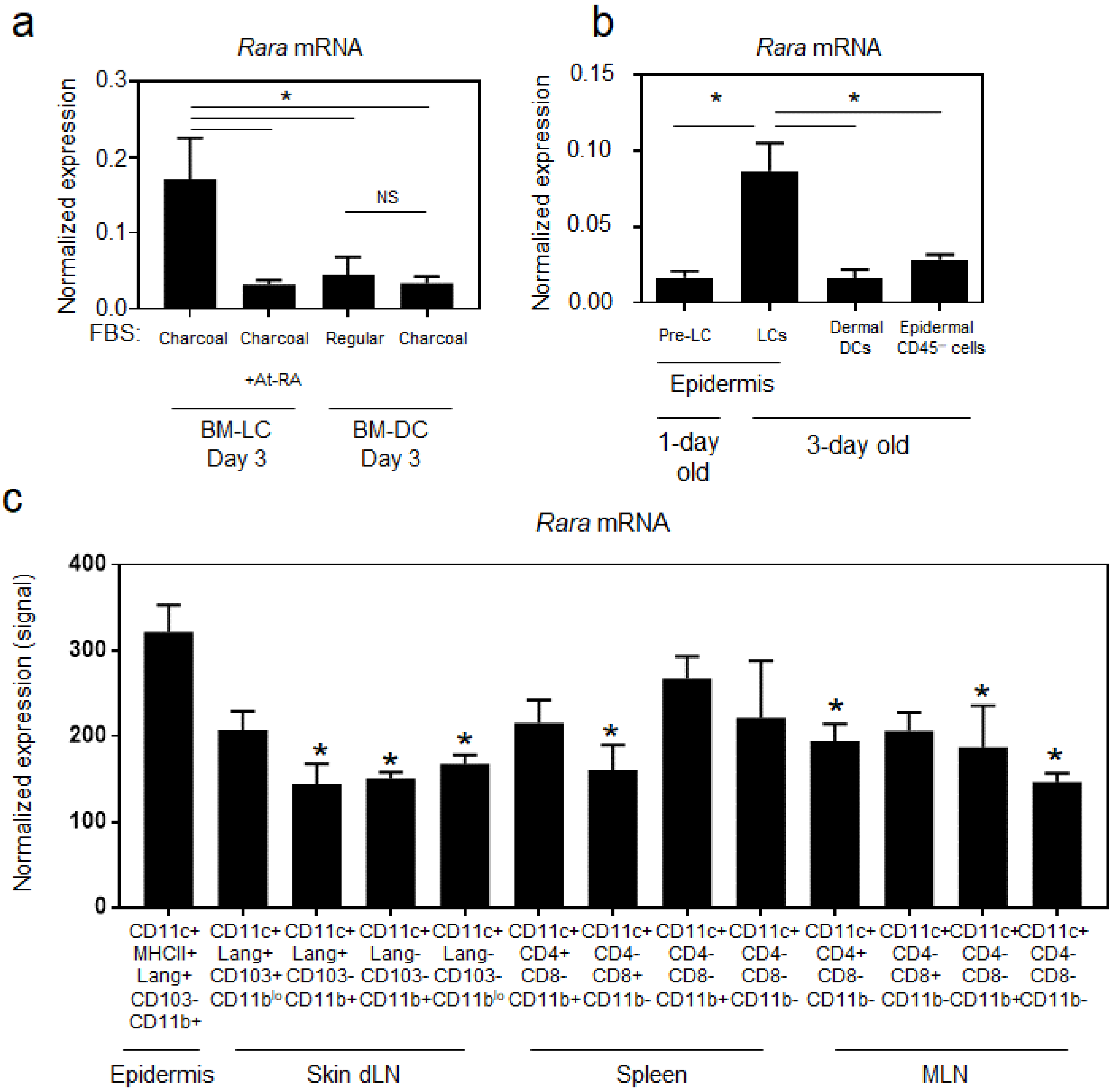

Supplementary Fig. 2. Expression of *Rara* mRNA by BM-LCs versus BM-DCs (a), primary CD11c$^+$ cells isolated from the epidermis and dermis of mouse pups (b), and primary DC subsets isolated from various tissues of adult mice (c). BM-LC cells were cultured for 3 days in complete RPMI-1640 medium supplemented with charcoal-treated FBS (10%), GM-CSF (20 ng/ml) and hTGF-β1 (10 ng/ml). At-RA was used at 10 nM. BM-DCs were similarly cultured for 3 days without using hTGF-β1. For primary cells (b), CD11c$^+$ cells were selected with magnetic beads (Mojosort^{TM}, BioLegend; average purity=90.1±0.9% for LCs, 90.3.9±0.4 for pre-LCs; 88.9±0.8% for dermal DCs; 95±0.8% for CD45-negative cells) from cell suspensions of epidermis of 1 or 3-day-old mouse skin. Affymetrix ST1.0 microarray data deposited in the Immgen database showing *Rara* expression by various DC subsets in indicated organs are shown (c). *Significant differences ($p<0.05$; n=3-8) by One-way ANOVA with Bonferroni corrections (a-c).

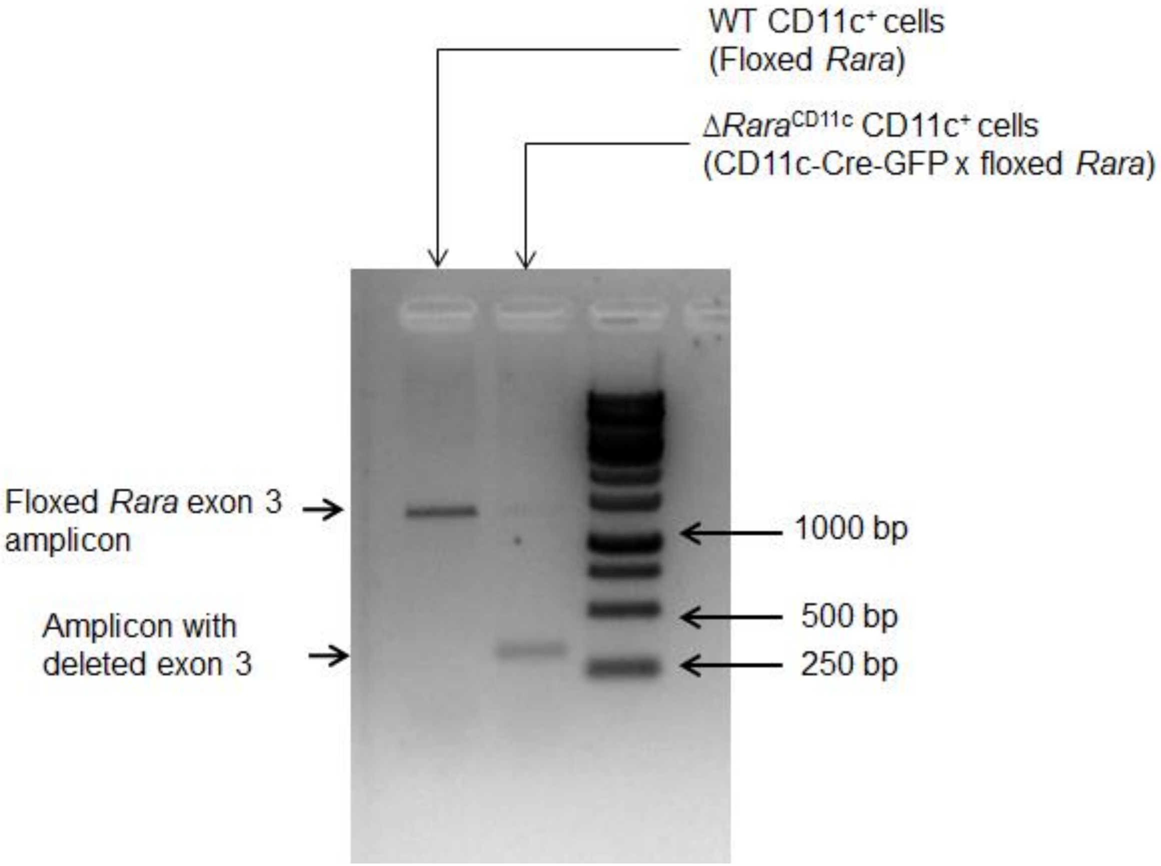

Supplementary Fig. 3. CD11c-Cre-GFP mice were crossed to a mouse line with floxed exon 3 of the *Rara* gene (NM_009024.2). Genomic DNA was extracted from BM-derived $CD11c^{+}$ cells cultured in GM-CSF (20 ng/ml), and PCR was performed with the primers shown in supplementary table 1.

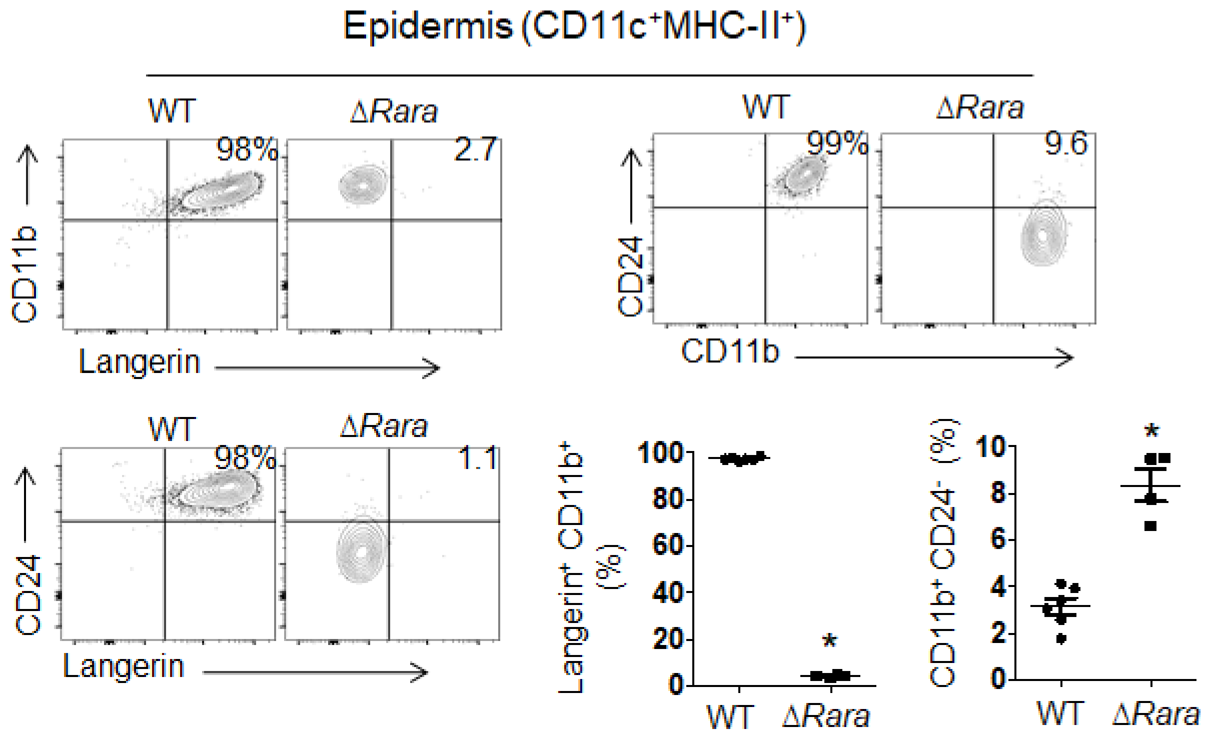

Supplementary Fig. 4. Phenotype of epidermal CD11c⁺ MHC-II⁺ cells in adult (5-8-week-old) WT versus *Rara*^{CD11c} mice. Expression of CD24, CD11b, and langerin by CD11c⁺ MHC-II⁺ cells was examined. Representative and combined data are shown (n=4-7). *Significant differences from WT by Mann-Whitney U test (p<0.05, unpaired, 2-sided).

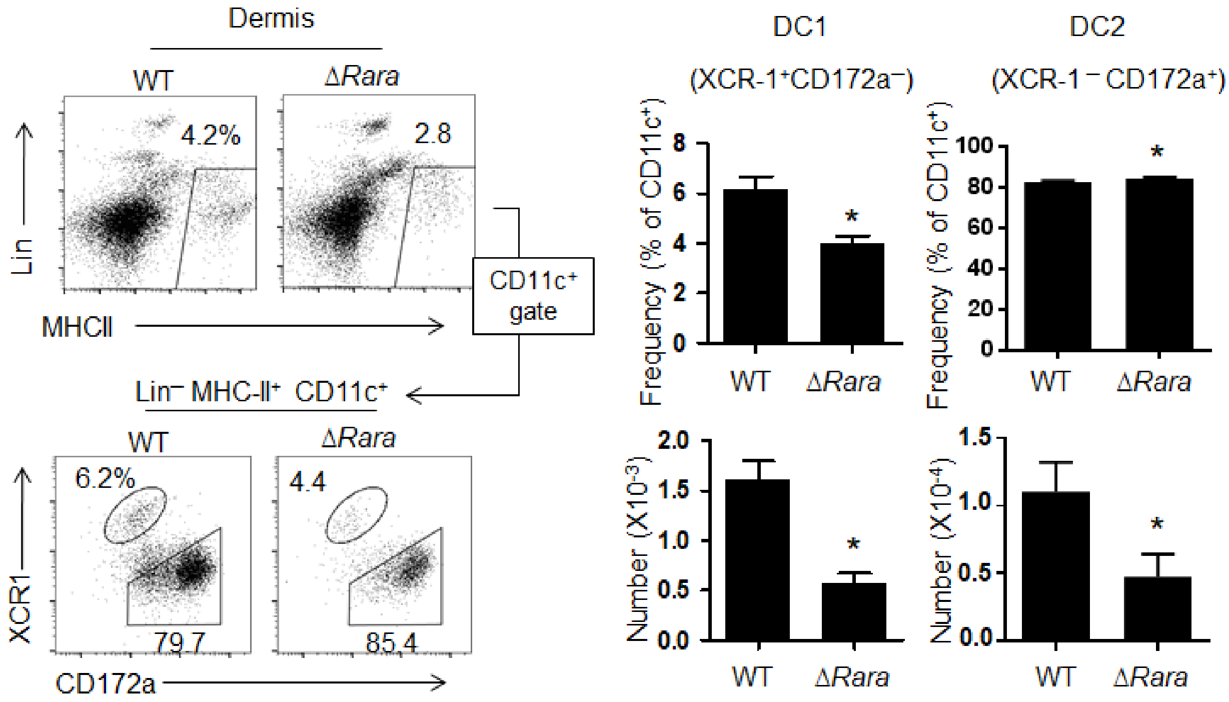

Supplementary Fig. 5. Dermal DC1 and DC2 subsets in adult WT versus *Rara*^{CD11c} mice. Frequencies and absolute numbers of DC subsets in WT versus Δ*Rara*^{CD11c} mice are shown in graphs. Representative and combined data from 3 independent experiments are shown (n=8). *Significant differences from WT by Mann-Whitney U test (p<0.05, unpaired, 2-sided).

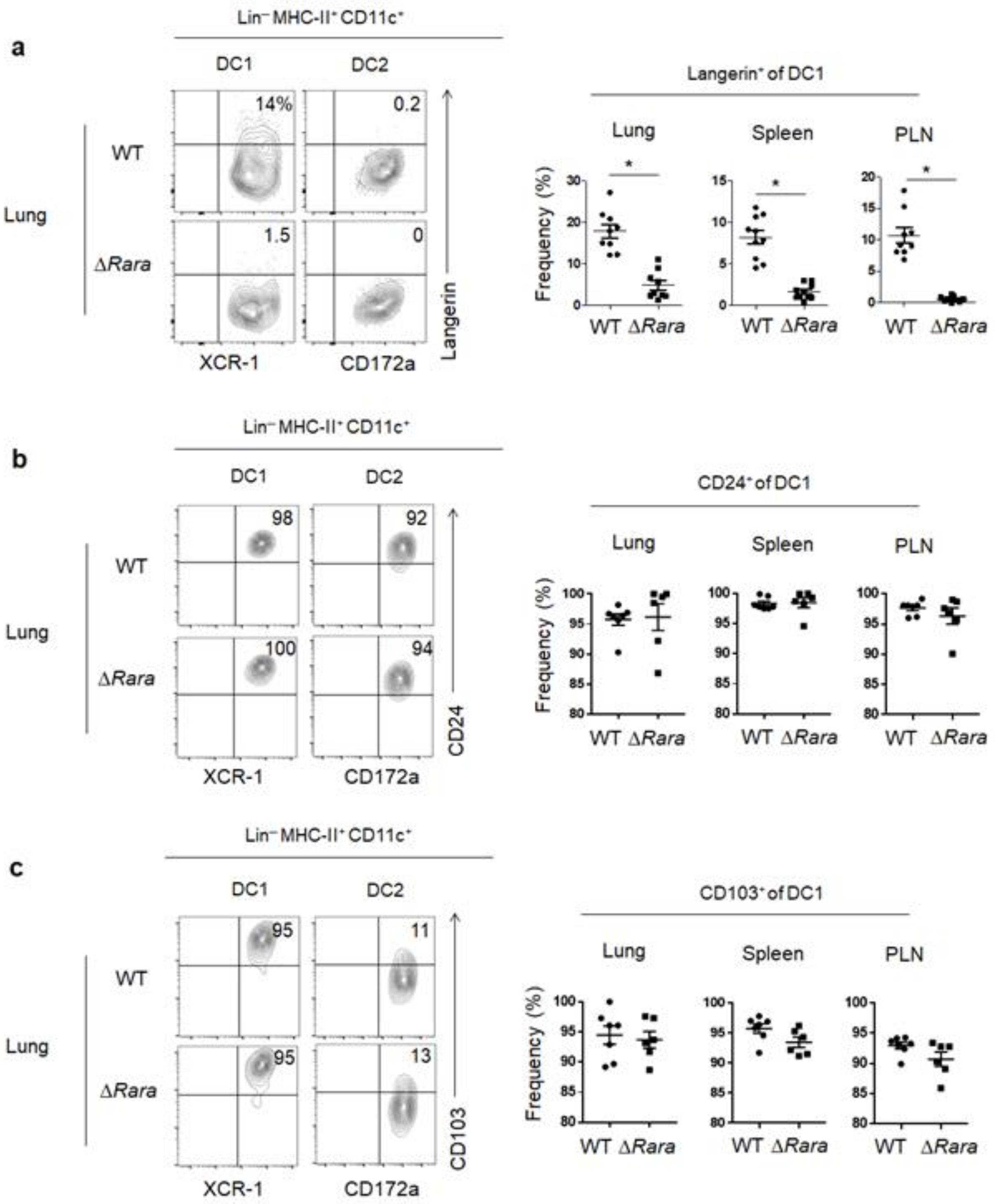

Supplementary Fig. 6. Expression of langerin (a), CD24 (b), and CD103 (c) by DC subsets in selected non-skin tissues of adult WT versus Δ*Rara*^{CD11c} mice. XCR1^{+} CD172a^{-} DC1 and XCR1^{-} CD172a^{+} DC2 subsets in lungs, spleen and PLN (inguinal and auxiliary) were examined for indicated antigens. Representative and combined data are shown (n=6-10). *Significant differences by Mann-Whitney U test ($p<0.05$, unpaired, 2-sided).

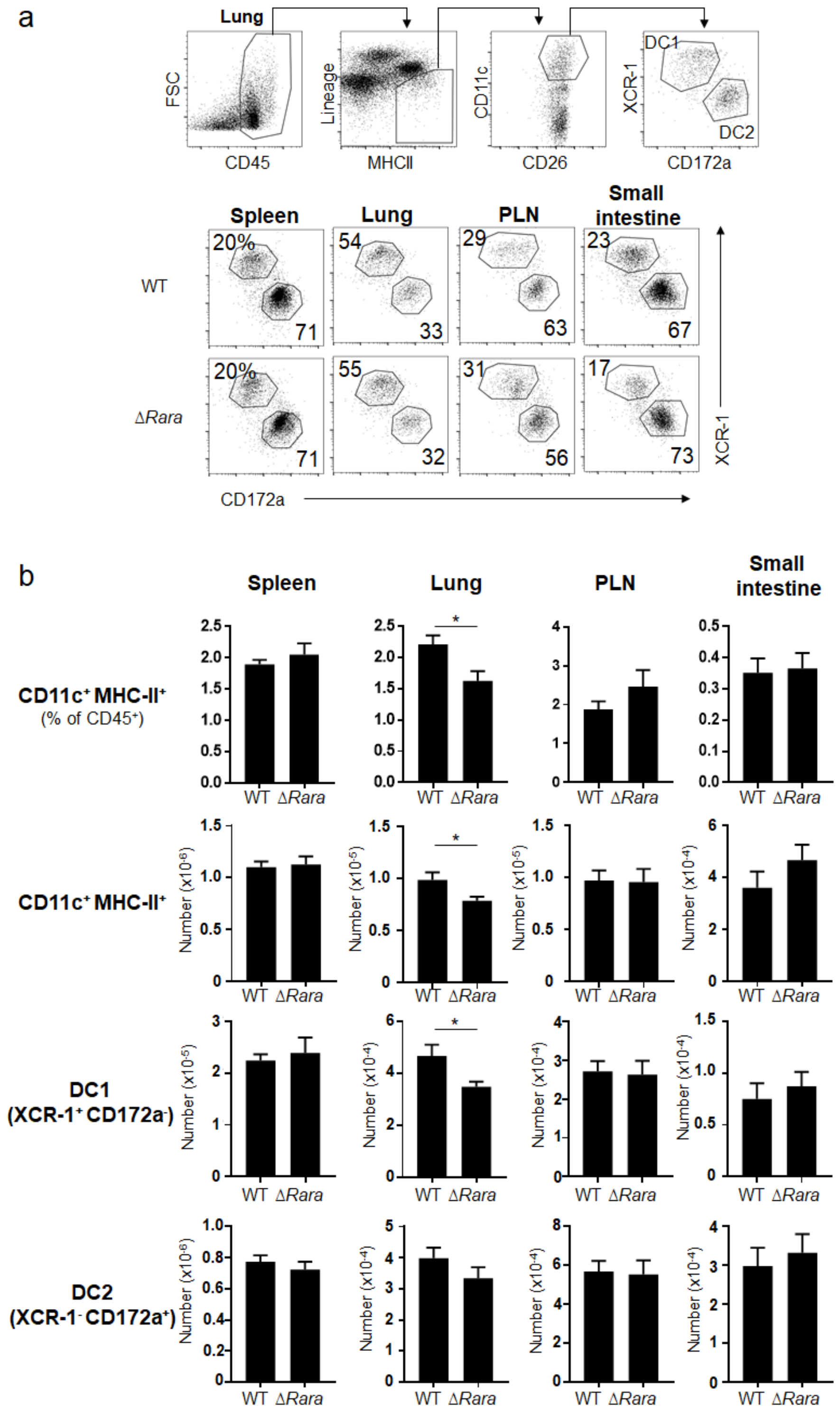

Supplementary Fig. 7. Frequencies and numbers of major DC subsets in non-skin tissues in adult WT versus Δ*Rara*$^{\mathbf{CD11c}}$ mice. (a) Gating strategy and representative dot plots. (b) The frequency and numbers of indicated CD11c^{+} MHC-II^{+} subsets. Representative and combined data are shown (n=9-16). *Significant differences by Mann-Whitney U test ($p<0.05$, unpaired, 2-sided).

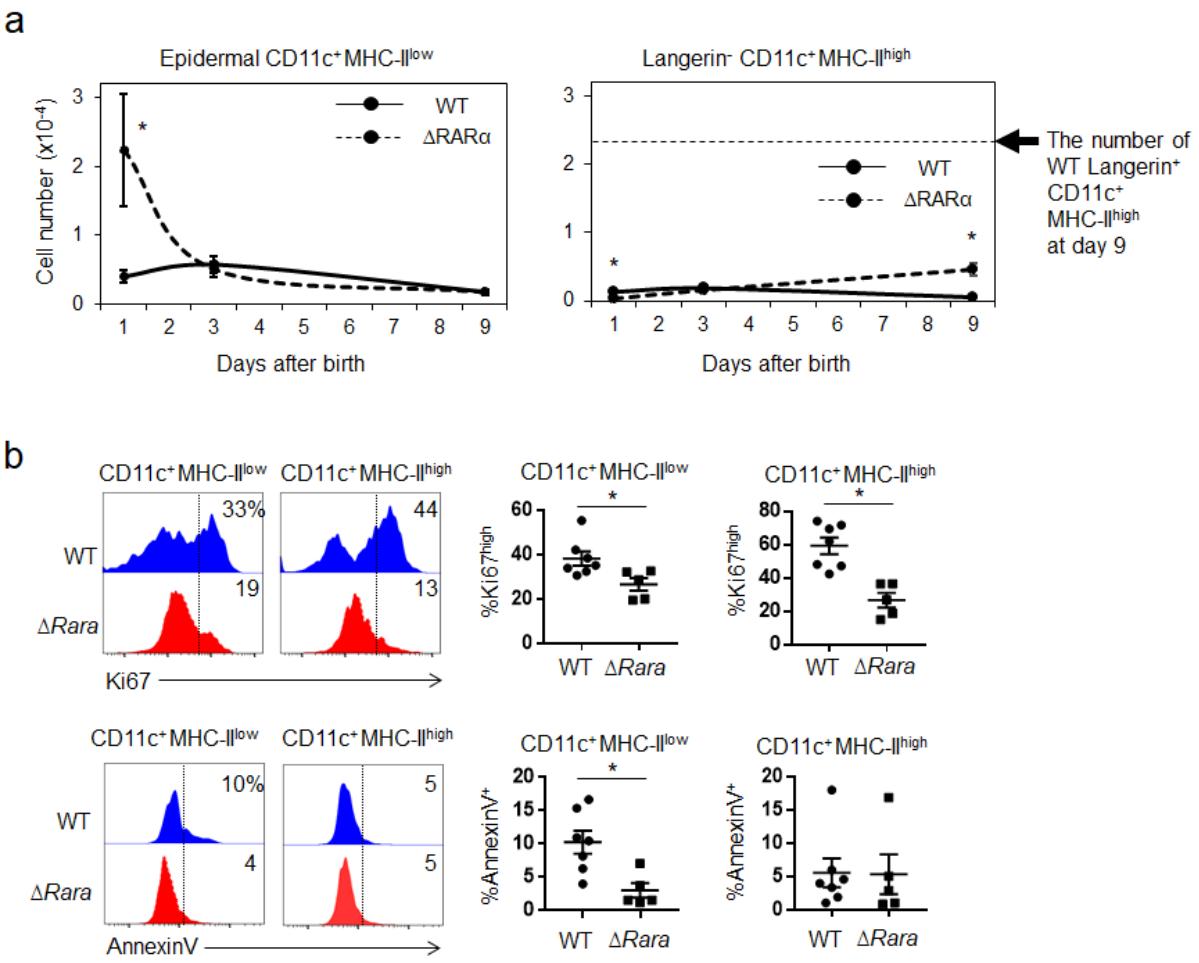

Supplementary Fig. 8. Population dynamics of CD11c^{+} MHC-II^{low} and CD11c^{+} MHC-II^{high} cells of newborn WT versus Δ*Rara*^{CD11c} mice. Representative and combined data are shown (n=9-16 for a; n=5-7 for b). Indicated cells in the trunk skin of WT and Δ*Rara*^{CD11c} mice was examined at indicated time points (a) or day 1 after birth (b). See Figure 2b for flow cytometry dot plots of these two CD11c^{+} cell populations in new-born mice. *Significant differences between the two groups by Mann-Whitney U test ($p<0.05$, unpaired, 2-sided).

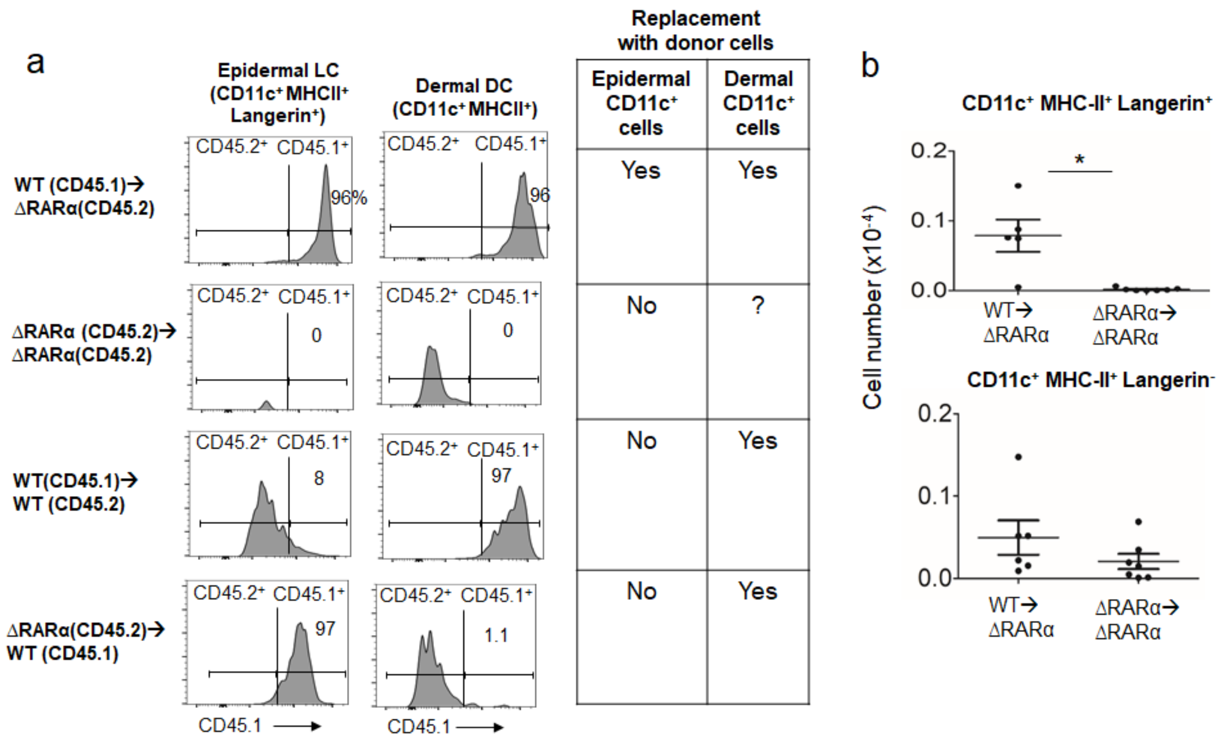

Supplementary Fig. 9. (a) A BM reconstitution study from WT or Δ*Rara*^{CD11c} mice into lethally irradiated WT or Δ*Rara*^{CD11c} mice. (b) Numbers of indicated langerin$^{+/-}$ CD11c^{+}MHC-II^{+} cells in the epidermis. The CD11c^{+}MHC-II^{+} cells were examined 12-15 weeks post-BM transfer. Please note that the LCs in WT recipients, which are radio-resistant, were not replaced by donor LCs. Representative and combined data (n=4-8) from at least 3 experiments are shown. Significant differences by Mann-Whitney U test (p<0.05, unpaired, 2-sided).

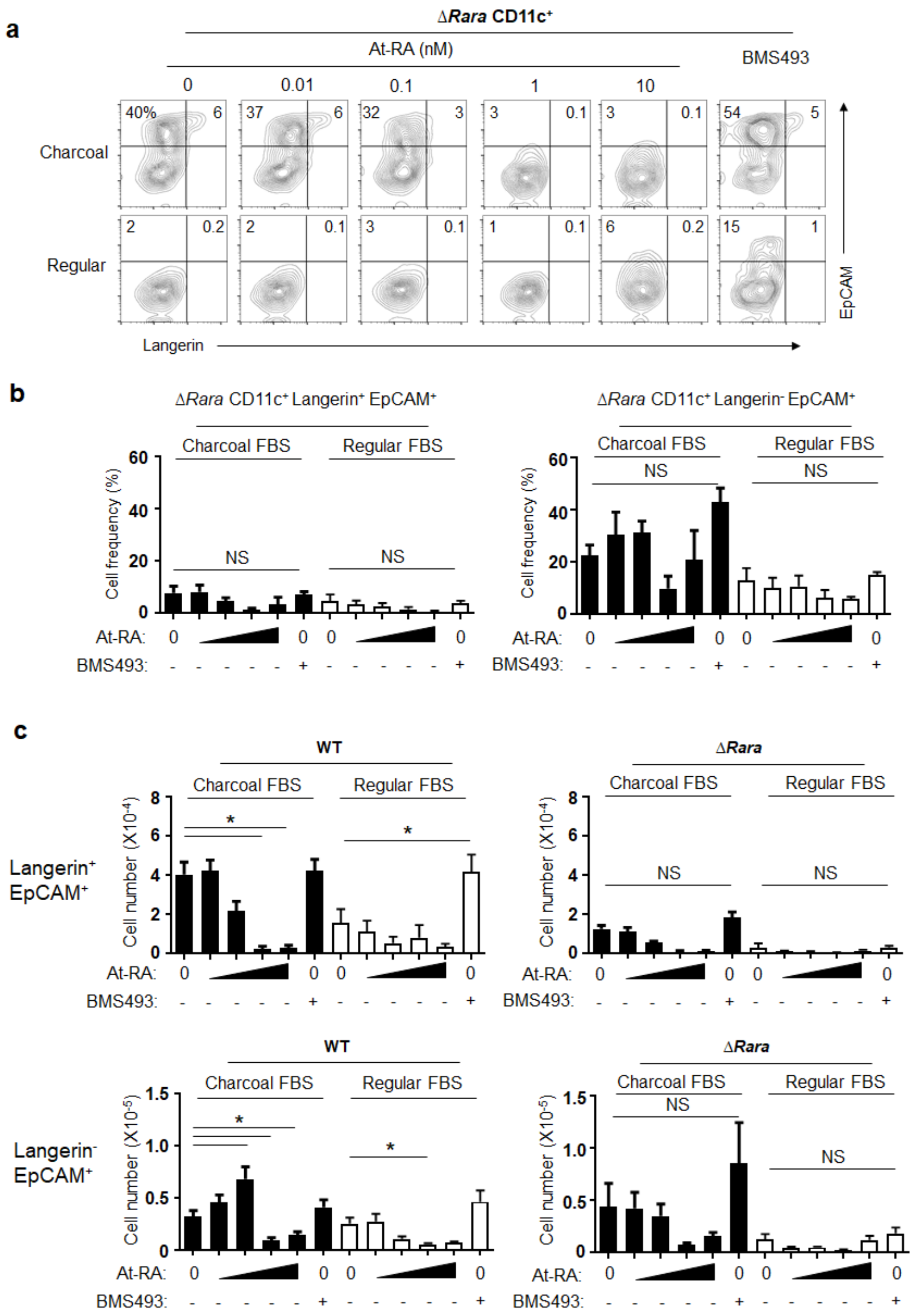

Supplementary Fig. 10. Impact of RA on Δ*Rara*$^{\mathbf{CD11c}}$ BM-LCs. (a) Δ*Rara*^{CD11c} BM cells were cultured with GM-CSF and TGF-β1 for 3 days in the presence of At-RA (0.01, 0.1, 1 and 10 nM) or BMS493 (100 nM) in media containing charcoal-treated or regular FBS. (b) Frequencies (% of CD11c^{+}) of indicated CD11c^{+} cells are shown. (c) Numbers of indicated WT and Δ*Rara*^{CD11c} CD11c^{+} cells are shown. *Significant differences by One-way ANOVA with Bonferroni corrections. NS, not significant.

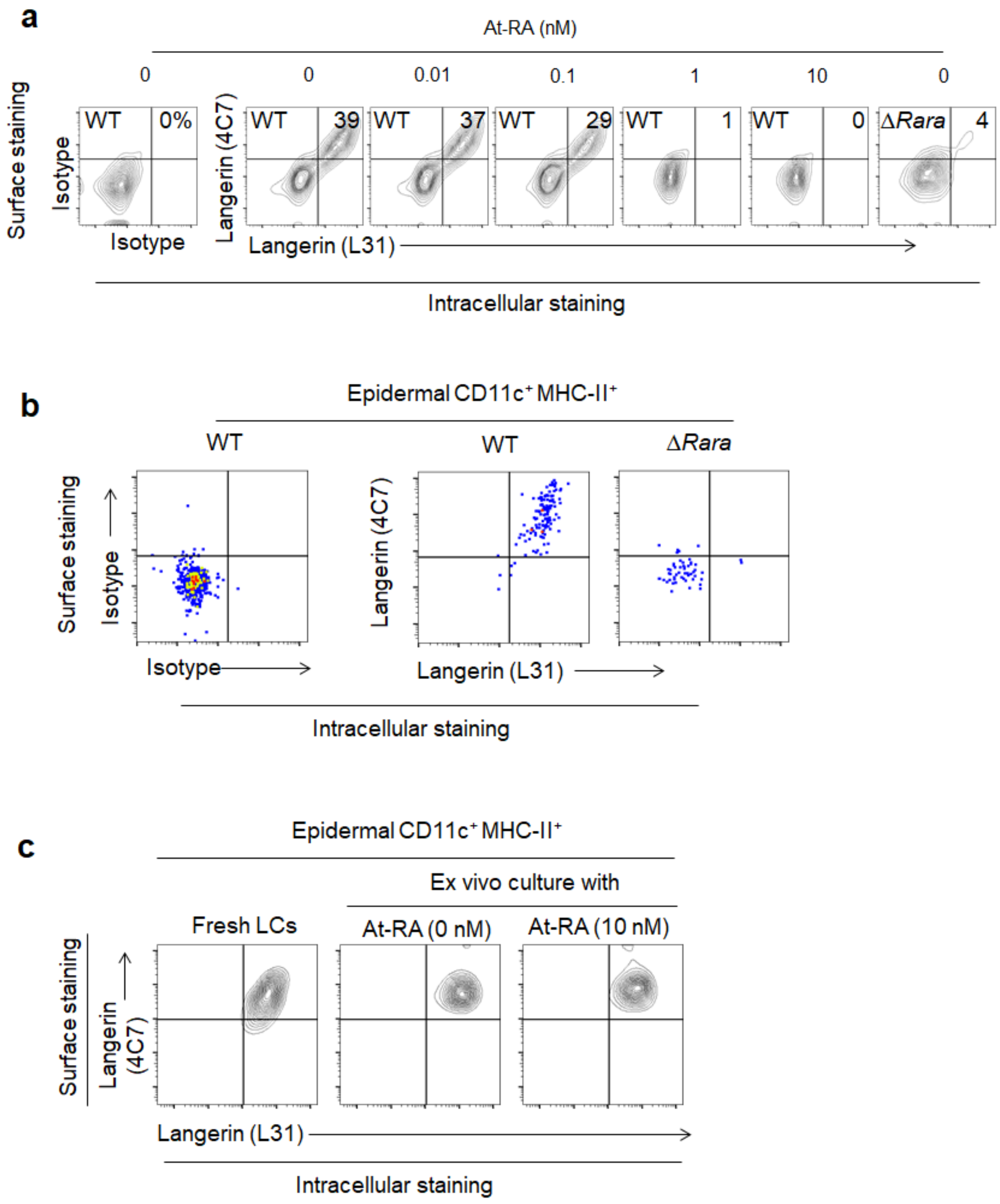

Supplementary Fig. 11. Effects of RA and RARα on the surface versus intracellular expression of langerin. (a) BM cells cultured in the BM-LC inducing condition for 3 days were examined by flow cytometry after staining for surface or intracellular langerin protein. Similarly, primary LCs were examined freshly (b) or after culture with At-RA (c). The cells were cultured in RPMI containing 10% charcoal FBS with or without At-RA (10 nM) for 24 h prior to surface and intracellular staining. Epidermal cells were isolated from adult ears and enriched with a 40%/70% Percoll gradient cut for leukocytes. Cells were first stained with antibodies for surface antigens, including langerin using APC-conjugated antibody (clone 4C7, Biolegend), followed by fixation and permeabilization and then stained for intracellular langerin using a PE-conjugated antibody (clone L31, eBioscience). Representative data from at least 3 independent experiments are shown.

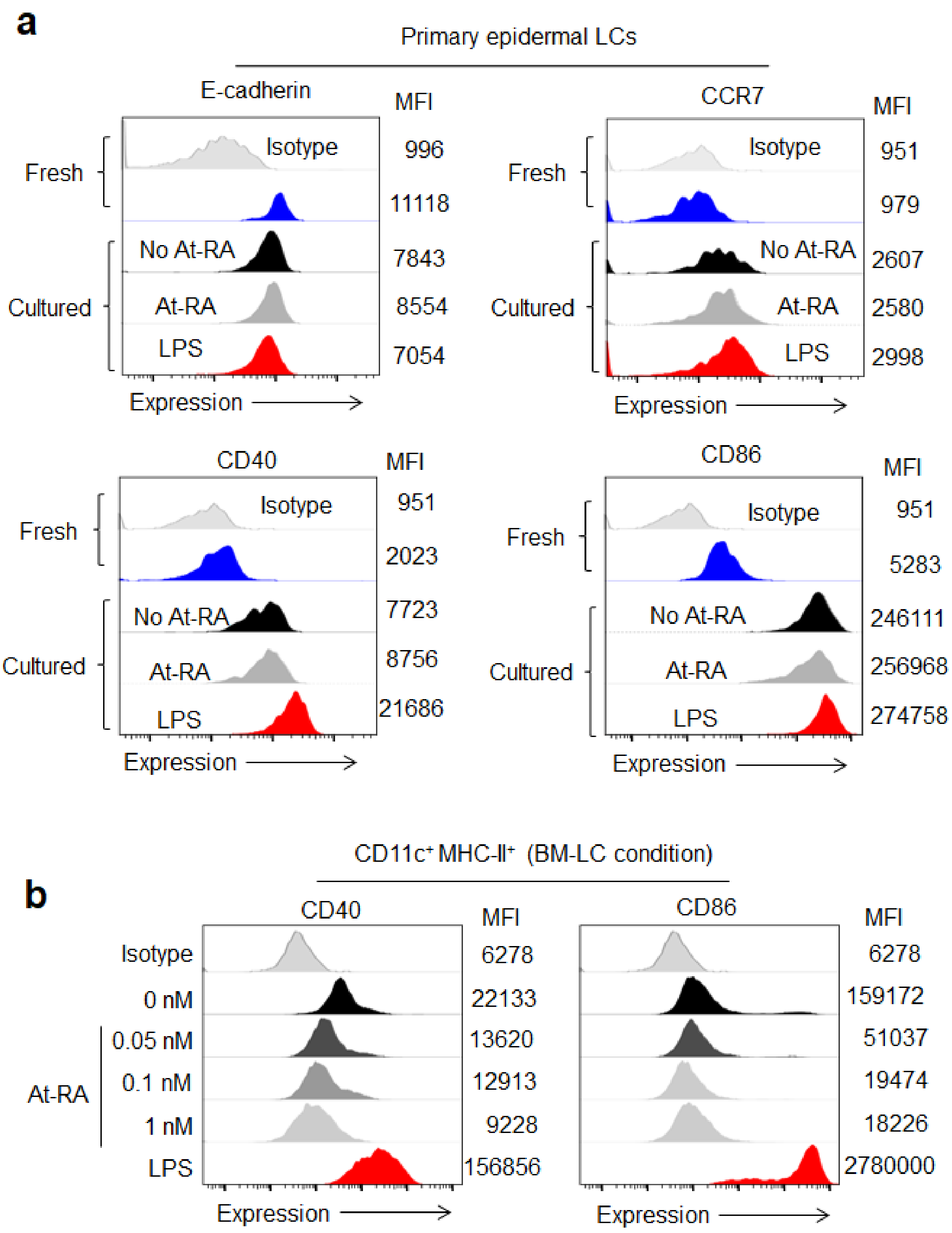

Supplementary Fig. 12. Effects of RA and RARα on maturation markers of primary LCs (a) and BM-LCs (b). (a) Effect of RA on primary CD11c$^+$ MHC-II$^+$ langerin$^+$ LCs. Epidermal leukocytes were cultured for 24 h without or with added RA (10 nM) and the expression of indicated antigens by CD11c$^+$ MHC-II$^+$ langerin$^+$ cells was examined. (b) Expression of CD40 and CD86 by CD11c$^+$ MHC-II$^+$ cells. BM cells were cultured with GM-CSF and TGF-β1 for 3 days. Mean fluorescence intensity (MFI) is shown. Lipopolysaccharide (LPS, 100 ng/ml) was used as a positive control. Representative data out of 3-4 independent experiments are shown.

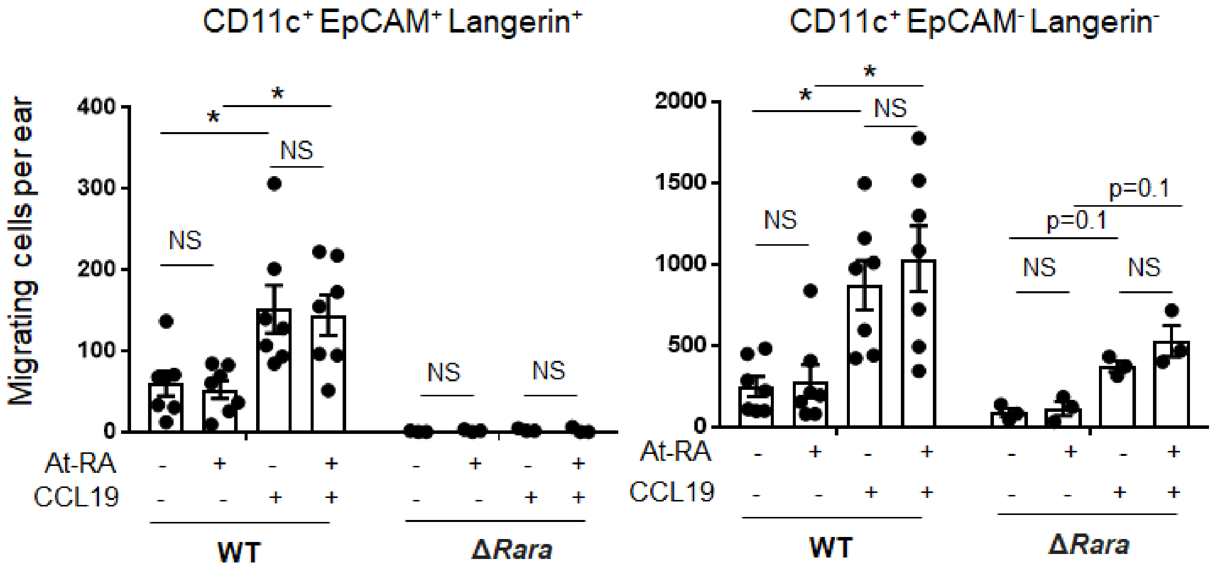

Supplementary Fig. 13. Effects of RA and RARα on emigration of langerin$^{+/-}$ CD11c^{+} MHC-II^{+} cells from ear explants. Indicated cells emigrating from ear explants from WT and Δ***Rara***$^{\mathbf{CD11c}}$ mice in response to At-RA (10 nM) and/or CCL19 (500 ng/ml) were counted by flow cytometry. The ear explants were prepared by separating the dorsal versus ventral halves and pre-incubating for 24 h in the same culture condition. Number of cells emigrating for the next 24 h were plotted. *Significant differences from respective controls by Mann-Whitney U test ($p<0.05$; n=3-7). NS, not significant.

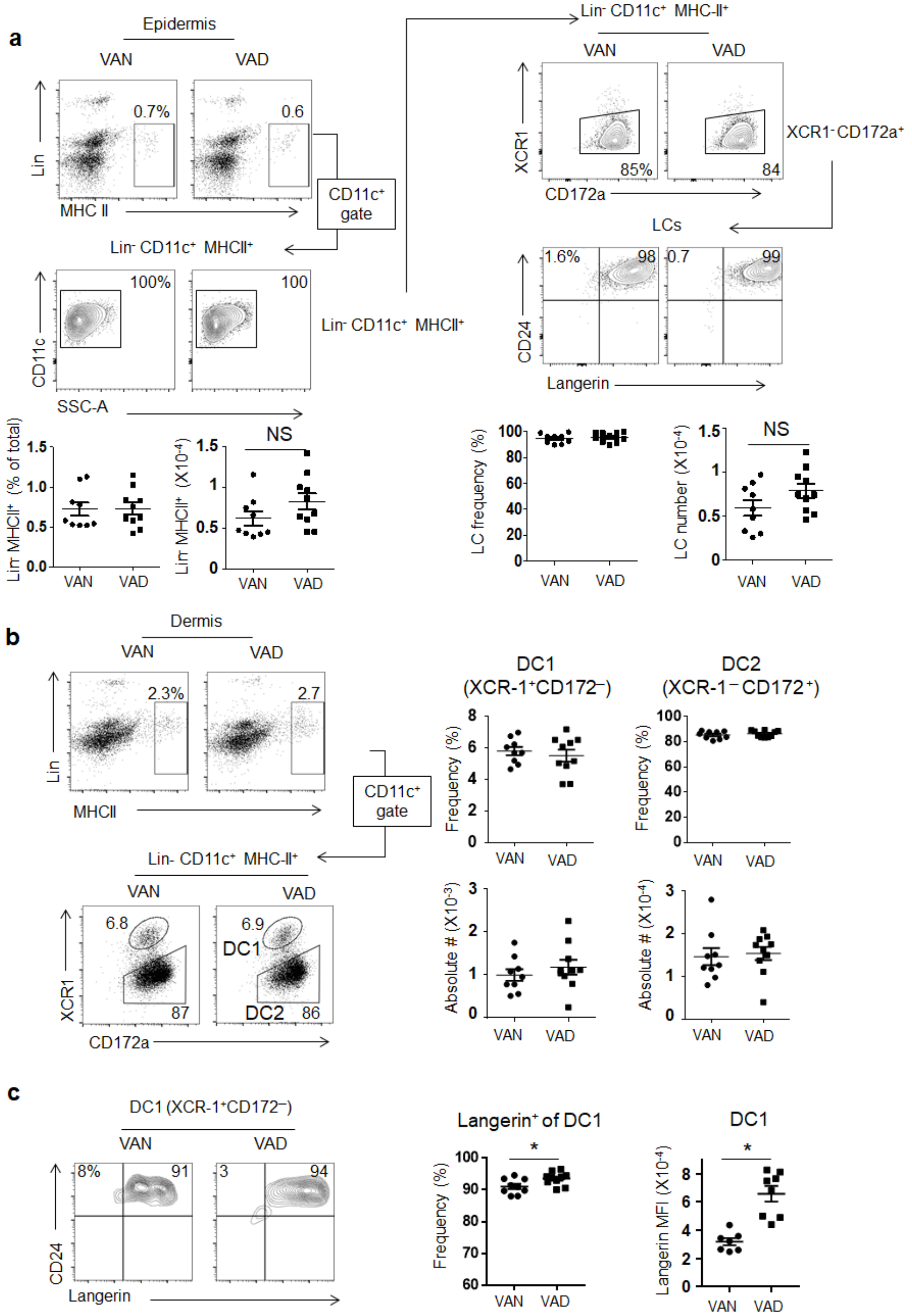

Supplementary Fig. 14. Impact of vitamin A deficiency (VAD) on langerin$^+$ DCs in the epidermis (a) and dermis (b, c) of adult mice. 10-12-week-old VAD and vitamin A normal (VAN) mice were examined for indicated DC subsets. Lineage-specific antibodies included CD3, NK1.1, CD19, B220, and CD64. Lin$^-$ MHC-II$^+$ CD24$^+$ CD11c$^+$ DC subsets that are either XCR1$^+$ CD172a$^-$ or XCR1$^-$ CD172a$^+$ were examined for frequency, cell number, and/or langerin expression. Representative and combined data are shown (n=9-10). *Significant differences from controls by Mann-Whitney U test (p<0.05, unpaired, 2-sided).

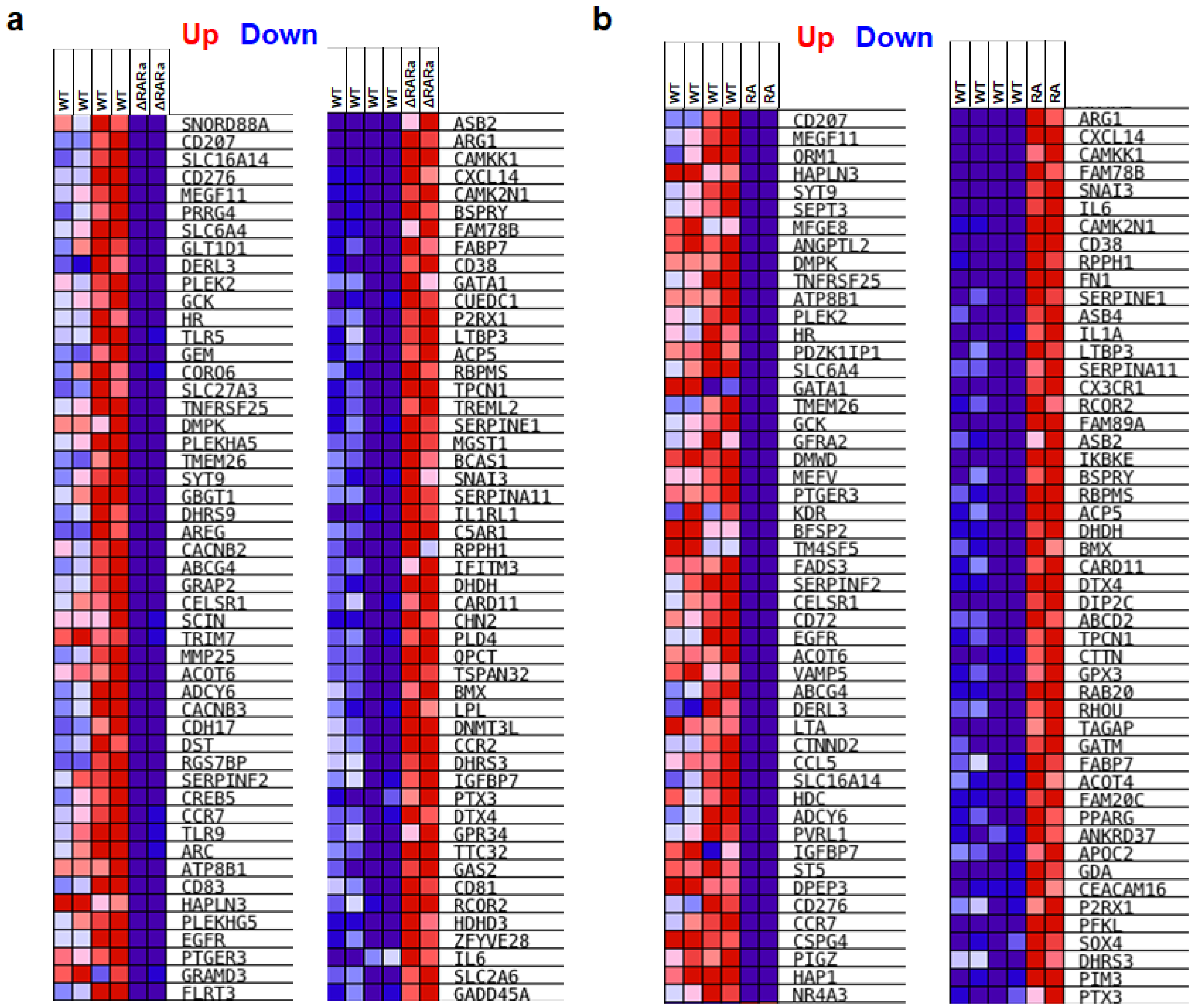

Supplementary Fig. 15. Expression of LC-associated genes and top 50 genes up or down-regulated in RARα deficiency or by At-RA. Heat maps, showing top 50 up (a) or down (b) -regulated genes in RARα deficiency or by At-RA, were generated by GSEA using log2 ratio of classes as a metric for ranking selected genes.

Supplementary Table 1: Primers used for qRT-PCR:

hCebpb-F TCGCAGGTCAAGAGCAAGG

hCebpb-R CTAGCAGTGGCCGGAGGA

hRunx3-F GACAGCCCCAACTTCCTCTG

hRunx3-R GGGGTTGGTGAACACAGTGA

mCebpb-F GTGGCCAACTTCTACTACGAGC

mCebpb-R GAGGCTCACGTAACCGTAGTCG

mRunx3-F CGA CTG CTA CTC CAA GCT CAA

mRunx3-R TCG GCT TCC ACG CCA TCAG

Rara-F: TCAGCCCCTCACCCTCCAAT

Rara-R: TACACTAACTACCCTTGACC)

mLangerin-F: CAACAGAGTGACATTCTGGAG

mLangerin-R: TTGGCACAGTGCTCATTGTTC
